# The integrated bioenergetic-interoceptive model of schizophrenia-spectrum disorders

**DOI:** 10.3389/fpsyt.2026.1911221

**Published:** 2026-10-30

**Authors:** István Szendi, Tamás Treuer

**Affiliations:** 1 Institute of Psychology, University of Szeged, Szeged, Hungary; 2 Department of Psychiatry, Kiskunhalas Semmelweis University Teaching Hospital, Kiskunhalas, Hungary; 3 Department of Psychiatry and Psychotherapy, Semmelweis University, Budapest, Hungary

**Keywords:** bioenergetics, gut-brain axis, inflammation, interoception, metabolic flexibility, predictive regulation, self-disorder

## Abstract

Schizophrenia has traditionally been conceptualized as a heterogeneous neurodevelopmental disorder involving neurotransmitter dysregulation, synaptic dysfunction, and large-scale brain network abnormalities. However, a growing body of convergent evidence indicates that schizophrenia is embedded in broader, system-level physiological dysregulation involving metabolic, immune, endocrine, gastrointestinal, autonomic, and interoceptive alterations. This article proposes an integrated bioenergetic-interoceptive model of schizophrenia-spectrum disorders, in which the disorder is understood as a disturbance of predictive allostatic regulation under energetic constraint. According to the model, reduced metabolic flexibility, altered cerebral bioenergetics, cumulative allostatic load, microbiome-gut-brain axis dysregulation, and disturbed interoceptive timing converge on late-maturing, energetically demanding association networks that support salience attribution, self-referential processing, cognitive control, social cognition, and embodied self-experience. Within this framework, the mismatch between energy demand and available capacity constitutes a functionally proximal energetic constraint on adaptive brain-body regulation, whereas impaired metabolic flexibility represents one possible dynamic mechanism underlying this constraint. Gut-immune-metabolite signaling is interpreted as a modulatory input system that may alter the reliability and metabolic cost of interoceptive inference. Gastric, cardiac, respiratory, and vagal rhythms may constitute candidate timing mechanisms through which bodily signals contribute to the temporal organization of the embodied self. The model further posits that neotenous association cortical systems and large-scale networks—including the default mode, salience, frontoparietal, cingulo-insular, and self-referential systems—are particularly vulnerable to convergent metabolic, inflammatory, autonomic, and interoceptive stressors. The model reframes schizophrenia as a multisystem disturbance of predictive brain-body regulation and delineates testable directions for future multimodal biomarker research and for adjunctive interventions targeting bioenergetic, inflammatory, autonomic, gut-brain, and interoceptive mechanisms.

## 1 Introduction

Schizophrenia is currently understood as a clinically heterogeneous, severe neurodevelopmental disorder that often follows a chronic course (1, 2). Its etiopathophysiology is complex and encompasses polygenic liability (3) as well as widespread cortical structural abnormalities (4). Functional dysconnectivity of large-scale brain networks is characteristic (5), together with disturbances of synaptic plasticity and characteristic abnormalities of neurotransmission (2). Cognitive impairments are detectable before the onset of psychosis and persist throughout the course of illness (6), whereas disturbances of basic self-experience show relatively selective, trait-like aggregation within the schizophrenia spectrum (1, 7). Pharmacological treatment continues to rely primarily on antipsychotics developed on the basis of neurotransmitter hypotheses (2), although their efficacy across the full symptom profile remains limited (8).

Established neurochemical, neurodevelopmental, and network models are indispensable, but on their own they do not fully specify how the affected neural systems are shaped by whole-organism energy regulation, immune and endocrine signaling, autonomic function, and cumulative environmental burden. In schizophrenia, neural dysfunction is embedded in whole-organism, multisystem brain-body dysregulation; understanding this dysregulation requires an integrated biological model. Large population databases indicate that multimorbidity, especially the clustering of cardiometabolic, respiratory, and endocrine diseases, is exceptionally high among people living with severe mental illness (SMI), particularly those with schizophrenia (SZ), bipolar disorder (BD), and recurrent major depressive disorder (MDD) (9, 10), and is a major determinant of markedly reduced life expectancy (11). Scientific advances over the past one to two decades have shown that these are not merely incidental comorbidities. Although medication, lifestyle, and other illness-related factors may contribute to these phenomena, certain metabolic abnormalities—particularly impaired glucose regulation and insulin resistance—have been detected in schizophrenia, for example, even before the initiation of antipsychotic treatment (12). In parallel, neuroimaging and molecular studies have identified abnormalities in cerebral energy metabolism (8). The co-occurrence of systemic and cerebral abnormalities provides a rationale for examining potentially interacting brain and body mechanisms within an integrated biological framework (13–15).

The maintenance of whole-organism homeostasis is now commonly understood within an allostatic framework. Allostasis denotes predictive regulation: the organism does not merely correct deviations after they occur through feedback mechanisms, but allocates energy in accordance with anticipated needs by means of feed-forward mechanisms.

Within allostatic theory, the brain responds to environmental stressors by modifying whole-body energy metabolism and immune activity, and, under challenge, it orchestrates a transition to a new homeostatic state—or, more precisely, a new homeodynamic state (16–18). Allostasis is not the operation of a single center or reflexive mechanism, but the product of a hierarchically organized anatomical network that supports prediction and feedback across multiple levels and is grounded in the cytoarchitectonic gradients of the cortex. The core of this network is formed by brainstem viscerosensory centers, especially the nucleus tractus solitarius (NTS) and the parabrachial complex, the hypothalamus, periaqueductal gray, the thalamic nuclear matrix (19, 20), and limbic-medial prefrontal regions (21, 22). These centers are connected through reciprocal loops to the cingulate cortex, the entire insular hierarchy, as well as the default mode and salience networks. The nodes of this network are responsible for organizing visceromotor efferent commands and integrating viscerosensory reafferents from the periphery (23). Gastrointestinal, cardiovascular, respiratory, and immune information is embedded in predictive regulation organized through this network, which provides the functional unity of the allostatic system. The gastrointestinal tract (GIT) is a central component of the allostatic architecture because food intake, nutrient utilization, and gut-immune interactions require continuous energy-allocation decisions. The most important nodes of the central representation of the GIT are the insula, as primary interoceptive cortex, and the limbic-medial network (24). In parallel, GI function is also influenced by top-down modulation. Enzymatic responses that prepare the intestine for digestion can be modified through classical conditioning. Higher cognitive and learning processes may build on such modulation, insofar as GIT-derived signaling dynamically shapes hippocampal function. Agranular limbic border regions, including the hippocampus and subiculum, emit centrifugal predictive signals that may function as allostatic goals, reference signals, or feed-forward models (24).

This article proposes a broad, organism-level model for understanding the phenomenology of schizophrenia. Its central claim is that early and persistent disturbances of brain metabolism, impaired metabolic flexibility, dysregulation of the microbiome-gut-brain axis, allostatic overload, and temporal disintegration of interoceptive rhythms are system-level biological processes relevant to understanding the complexity of the disorder.

## 2 Methodological approach to the literature synthesis

This manuscript is a narrative-theoretical synthesis whose aim is model construction: the formulation of a testable integrative framework that connects schizophrenia with organism-level energy regulation and predictive brain-body dynamics. Accordingly, the literature was not selected to provide exhaustive coverage of every mechanism in schizophrenia, but to examine domains that are theoretically central to the proposed model: metabolic flexibility and cerebral bioenergetics; allostasis and predictive regulation; the microbiome-gut-brain axis; interoceptive oscillations and bodily rhythms; the neotenous association cortex; and large-scale network neurobiology in schizophrenia. The literature used for this synthesis was identified primarily through searches of the PubMed and Scopus databases. The search strategy focused on intersections of schizophrenia-spectrum disorders with the following keywords: “metabolic flexibility,” “allostatic load,” “gut–brain axis,” “vagal tone,” and “interoception.” Where direct evidence from schizophrenia-spectrum samples was unavailable, findings from diagnostically broader samples with first-episode or non-affective psychosis, psychosis-spectrum studies, or transdiagnostic investigations were used as indirect support and identified accordingly. To reduce selective argumentation, the synthesis prioritizes convergent evidence across levels of analysis, including population health data, metabolic findings in drug-naive samples, neuroimaging and molecular evidence on cerebral bioenergetics, developmental cortical vulnerability, allostatic neuroanatomy, and gut-immune-endocrine signaling. Competing interpretations are treated not as mutually exclusive alternatives, but as constraints on model building. The argument therefore distinguishes established findings from mechanistic extrapolations: systemic metabolic abnormalities and treatment limitations are presented as empirical starting points, whereas the proposed integration of metabolic inflexibility, allostatic overload, gut-brain dysregulation, and interoceptive desynchronization is advanced as a hypothesis for future investigation.

## 3 Conceptual framework: predictive allostatic regulation under energetic constraint

This section presents the conceptual architecture of the proposed model and the mechanistic hypotheses linking its components; the detailed empirical basis of each component is presented in Section 4, while **Supplementary Table S1** summarizes the origin and type of the evidence, the population studied, treatment status, diagnostic scope and directness, and the principal limitations. Empirical starting points are identified below with representative citations, whereas relationships not yet directly tested are formulated as hypotheses derived from the present model. **Figure 1** illustrates the proposed relationships among the model components, and **Table 1** summarizes their roles and evidential status.

**Figure 1:**
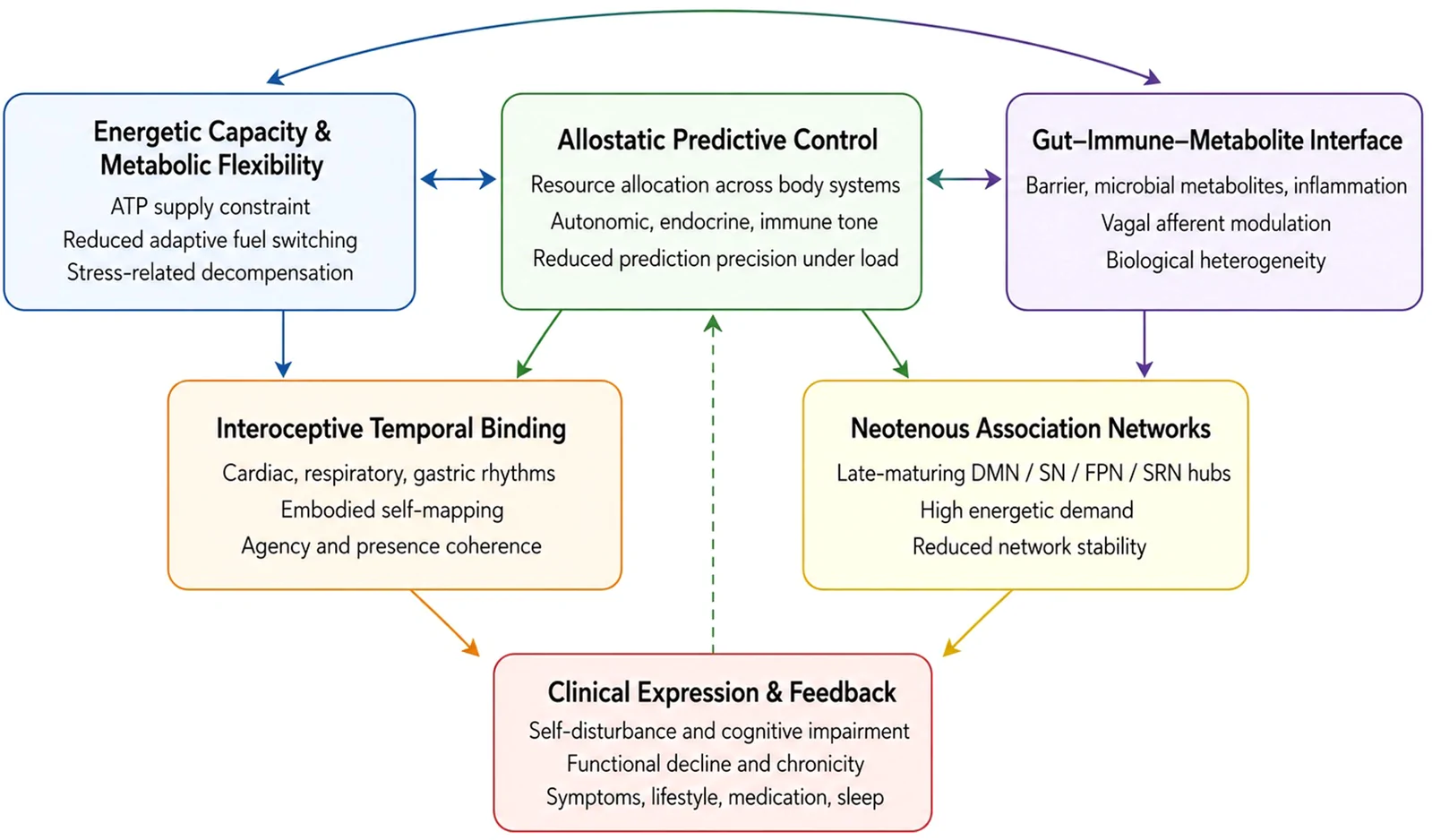
The integrated bioenergetic–interoceptive model of schizophrenia-spectrum disorders. Solid arrows indicate the proposed directional or reciprocal relationships among model components, whereas the dashed arrow indicates feedback from clinical expression to allostatic predictive control. DMN, default mode network; SN, salience network; FPN, frontoparietal network; SRN, self-referential network. In the present model, SRN is used as a functional designation for self-referential processing and should not be interpreted as a standardized, anatomically distinct subnetwork of the DMN. The association-network component does not imply that all listed regions are neotenous or disproportionately expanded. “High energetic demand” is a model hypothesis, not an established property of every region; elevated regional aerobic glycolysis alone does not establish increased total energy expenditure (28). The detailed functional architecture and evidential basis of the individual layers of the model are presented in **Supplementary Table S1**.

**Table 1:** The integrated bioenergetic–interoceptive model of schizophrenia-spectrum disorders.

Model component	Role in the model	Evidential status
Energetic capacity and metabolic flexibility	Energetic constraint is defined as a mismatch between current demand and available capacity. Impaired metabolic flexibility is one candidate dynamic mechanism through which this constraint may become apparent, particularly under challenge.	Moderate-to-strong convergent evidence for peripheral and cerebral bioenergetic abnormalities; limited direct evidence for impaired dynamic substrate or pathway switching.
Predictive allostatic regulation	Anticipatory allocation of metabolic, autonomic, endocrine, and immune resources; a candidate bridge between systemic physiological state, interoceptive precision, and prediction-error weighting.	Moderate direct evidence for elevated cumulative multisystem allostatic load; integrative and theory-based evidence for the proposed energetic–interoceptive mechanism.
Gut–immune–metabolite interface	A peripheral modulatory system involving intestinal barrier function, microbial metabolites, inflammatory signaling, and vagal afferents; may alter the reliability and energetic cost of bodily signals.	Moderate but heterogeneous human evidence, including some medication-free and drug-naïve findings; mechanistic specificity and temporal precedence remain unresolved.
Interoceptive temporal binding	Cardiac, respiratory, and gastric rhythms are candidate timing signals for embodied self-representation; the insula links viscerotopic representations with salience processing and may also serve as a neuroimmune interface.	Moderate evidence for autonomic-vagal dysfunction; sparse direct evidence from schizophrenia-spectrum samples for gastric-brain coupling and insular timing; hypothesis-generating with respect to self-disorder.
Neotenous association networks	Selected late-maturing association networks, including regions within the default mode, salience, and frontoparietal networks, are proposed as potential convergence sites for interacting developmental, metabolic, and interoceptive influences.	Strong supporting evidence from developmental and schizophrenia neuroimaging; the proposed cross-level convergence with systemic dysregulation remains inferential.
Clinical expression and feedback	Self-disorder, cognitive and salience-processing dysfunction, functional decline, and somatic burden are emergent outcomes; symptoms, sleep, lifestyle, medication, and social disadvantage may feed back on regulatory load.	Strong evidence for the clinical and multisystem burden; the direction and strength of feedback loops require longitudinal testing.

Evidence ratings are qualitative and are not formal GRADE classifications. For the provenance, diagnostic scope, study type and population, treatment status, representative references, and limitations of the evidence supporting each component, see **Supplementary Table S1**. DMN, default mode network; SN, salience network; FPN, frontoparietal network; SRN, self-referential network.

We hypothesize that, in the pathogenetic process leading to schizophrenia, metabolic inflexibility, gut-immune-metabolite signaling, and altered interoceptive timing converge on developmentally late-maturing, energetically demanding association networks, thereby impairing self-experience, salience processing, and cognition. We propose that one possible integrative interpretation of schizophrenia involves disturbances in predictive allostatic regulation under energetic constraints. Within this framework, reduced metabolic flexibility, increased allostatic load, and altered processing of peripheral interoceptive signals may represent interrelated aspects of dysregulated brain–body coordination.

A mismatch between current energy demand and available capacity may constitute an energetic constraint; limited substrate availability, mitochondrial ATP production, phosphotransfer buffering, or neuron-glia metabolic coordination may each contribute to this constraint. Metabolic flexibility is a narrower concept, denoting the capacity to adaptively switch between substrates and energy-producing pathways in response to changing demands. In our theoretical framework, the energetic capacity constraint is understood as a functionally proximal, but not necessarily etiologically primary, condition: it may influence the magnitude of allostatic, cognitive, immunological, and interoceptive demands for which the brain-body regulatory system can compensate. We regard impaired metabolic flexibility as one possible dynamic mechanism underlying this capacity constraint; its plausibility is supported by peripheral and cerebral bioenergetic abnormalities demonstrated in schizophrenia (2, 12, 25–27). The model’s relevance to the schizophrenia spectrum derives not from energetic constraint per se, but from its specific configuration with developmentally vulnerable association networks, allostatic dysregulation, and disturbances of embodied self-experience. If neuronal and glial systems have limited capacity to adapt the utilization of energy substrates, including glucose, lactate, and ketone bodies, to regulate ATP production, and to maintain high-energy phosphate transfer and buffering in response to changing demands, higher-order cortical networks may become more vulnerable when energetic demand exceeds available capacity. Certain late-maturing heteromodal association networks may be particularly relevant to this hypothesis because of their prolonged developmental trajectories and functional complexity. Some of these late-maturing heteromodal association networks overlap with cortical regions that exhibit elevated aerobic glycolysis in healthy individuals (28). Although this spatial overlap does not by itself establish increased total energy expenditure or greater vulnerability to energetic constraints in schizophrenia, its potential relevance to bioenergetic vulnerability warrants direct investigation.

Elevated allostatic load has been reported in first-episode psychosis and during the early stages of schizophrenia (29, 30). Within this architecture, we posit allostatic dysregulation as one possible bridge between peripheral physiological states and psychopathological outcomes. Increased allostatic load, chronic stress exposure, inflammatory signaling, autonomic imbalance, and altered metabolic state may distort interoceptive precision and the weighting of prediction error. These factors do not produce symptoms through a single linear causal pathway; instead, they may gradually reduce the flexibility of predictive brain-body regulation, thereby increasing instability in salience attribution, reducing cognitive efficiency, and heightening the vulnerability of embodied self-experience.

In the present framework, immune-inflammatory signaling is not assigned to a single model layer; rather, it may act as a cross-level modulator of energetic demand, allostatic resource allocation, signals originating at the intestinal barrier, autonomic regulation, and the precision of interoceptive inference.

Human studies in schizophrenia have reported alterations in microbiome composition and functional potential, as well as in barrier-related and immunometabolic signaling, although the findings are heterogeneous and may be confounded by numerous clinical, lifestyle, and treatment-related factors (31–33). According to our model, these alterations may modify the reliability and metabolic cost of interoceptive regulation. Dysbiosis, altered intestinal permeability, immune activation, short-chain fatty acid signaling, tryptophan-kynurenine metabolism, and vagal afferent pathways may influence central predictive regulation by altering inflammatory tone, metabolite availability, autonomic balance, and the reliability of signals arising from the body. The present model therefore treats gut-brain abnormalities as plausible contributors to allostatic and interoceptive instability, while emphasizing that human evidence remains limited and heterogeneous.

A further, more hypothesis-generating component concerns interoceptive and gastric-brain timing. Stomach-cortex coupling, cardiac afferent signaling, respiratory rhythms, and vagal timing may contribute to the temporal organization of embodied self-experience. To date, gastric-cortical coupling and the stomach-linked resting-state brain network have been demonstrated primarily in studies of healthy participants (34, 35), whereas direct evidence in schizophrenia is currently largely confined to autonomic/vagal and gastrointestinal functional abnormalities (36–38). On this basis, in the present model, disturbed interoceptive timing is conceptualized as a candidate mechanism through which metabolic, autonomic, and gut-derived disturbances may influence subjective embodiment and the coherence of self-experience.

Developmental studies have documented prolonged maturation of selected association cortical regions, while metabolic imaging in healthy individuals has demonstrated regionally elevated aerobic glycolysis in parts of the prefrontal and parietal cortices (28, 39, 40). Structural neuroimaging in schizophrenia has identified cortical thinning, particularly in frontal and temporal regions (4). In typically developing youth, higher schizophrenia polygenic risk has been associated with greater thickness in association cortex, whose spatial distribution overlaps cortical thinning patterns in schizophrenia (41). Within the present model, the possibility that developmental characteristics and regional metabolic specialization may contribute to vulnerability under energetic constraints, particularly in the presence of interacting inflammatory, autonomic, and interoceptive disturbances, constitutes a hypothesis requiring direct empirical investigation. One hypothesized central point of convergence in the present model is the reduced stability of these developmentally protracted, energy-demanding association networks. These late-maturing, evolutionarily expanded cortical systems support self-referential processing, abstraction, mentalizing, salience integration, and cognitive control. Because of their prolonged developmental trajectories and high energetic requirements, they are plausible targets of convergent metabolic, inflammatory, autonomic, and interoceptive stressors. The resulting phenotype would be expected to include not only positive psychotic symptoms, but also disturbances of self-experience, salience dysregulation, cognitive impairment, and vulnerability to stress-related psychosis.

## 4 Evidence domains

We review five evidence domains in order to distinguish direct empirical findings from mechanistic inferences and more speculative model extensions. The aim is to organize convergent evidence supporting a multisystem interpretation of schizophrenia. **Supplementary Table S1** summarizes the empirical basis of each model component; the type of evidence, study population, diagnostic scope, and qualitative strength of the evidence; and mechanistic inferences not yet directly tested.

### 4.1 Metabolic inflexibility

A growing body of research has identified alterations in cerebral bioenergetic processes in schizophrenia, although their pathophysiological significance and causal relationship to the disorder remain to be established. Synthesizing data and mechanisms, Sarnyai and Ben-Shachar (2) proposed the hypothesis of “impaired dynamic metabolic flexibility,” according to which the brains of individuals with schizophrenia show an impaired ability to switch among energy substrates, especially glucose and ketone bodies, in accordance with changing cellular demands. In their theory, genetic vulnerability, environmental exposures, and stress converge on critical cellular energy-metabolic mechanisms and reduce the capacity of neurons to produce sufficient ATP during periods of increased energy demand. This energetic deficit may lead to synaptic dysfunction and neural network instability, especially in brain regions with high metabolic demands, and may contribute to the emergence of cognitive deficits and other clinical symptoms.

Peripheral metabolic data primarily point to disturbances of glucose metabolism (2). During the first psychotic episode, patients with schizophrenia often show elevated blood glucose levels and insulin resistance even before pharmacological treatment is initiated (12). For lactate, the strongest meta-analytic evidence concerns elevated brain lactate levels, detectable in post-mortem and *in vivo* studies even when blood lactate levels do not differ meaningfully from those of healthy controls (42). In addition, reduced glucose uptake and glucose hypometabolism have been detected in the brain, particularly in frontal cortical regions, with large effect sizes, although the magnitude of these abnormalities varied according to illness stage and medication status (26). Magnetic resonance spectroscopy studies in schizophrenia, again primarily in frontal regions, indicate dynamic bioenergetic disturbance, particularly impaired creatine kinase-mediated phosphate transfer, which is detectable not only in chronic schizophrenia but also in first-episode psychosis (25, 43). Cellular studies in schizophrenia point to mitochondrial/bioenergetic abnormalities, particularly changes in mitochondrial complex I expression and function (44), broader changes in oxidative phosphorylation (OXPHOS) complex activity and gene expression (45), and, with weaker evidence, alterations in mitochondrial morphology and dynamics (46).

Recent human postmortem, cell type-resolved, and induced pluripotent stem cell (iPSC)-based studies suggest that the spatial and cellular manifestations of schizophrenia-associated mitochondrial and OXPHOS abnormalities may be heterogeneous, while some abnormalities also converge across multiple brain regions or cortical layers (47–52). Disease-independent human brain-mapping data likewise show marked regional, cell-type, laminar, and age-related differences in the organization of cerebral energy-production pathways, thereby providing a biological basis for context-dependent bioenergetic vulnerability (53, 54). On this basis, we regard region- and cell type-specific metabolic inflexibility as a testable, context-dependent form of load-dependent bioenergetic vulnerability.

### 4.2 Predictive allostatic regulation and stress-related reallocation of energetic resources

Allostasis is anticipatory regulation aligned with predicted internal and environmental demands and entails the advance allocation of physiological and energetic resources (23, 55, 56). We distinguish this from allostatic load, the cumulative multisystem cost of repeated or sustained adaptation, and from allostatic overload, in which demands exceed adaptive capacity and may lead to pathophysiological consequences. We therefore interpret predictive allostatic regulation as a central organizing principle of the model that may link challenges arising from the internal and external environments to metabolic, autonomic, endocrine, immune, and interoceptive states.

During an acute challenge, priorities in energy use and physiological regulation are temporarily reorganized. We hypothesize that, under repeated or sustained demand and limited metabolic flexibility, the energetic cost of allostatic responses may increase, post-challenge recovery may become inadequate, and regulatory reserve may become constrained. We posit a reciprocal relationship: limited energetic adaptability may make allostatic responses more costly, whereas accumulating allostatic load may further diminish metabolic reserve. Building on the theoretical relationship between allostasis and interoceptive inference and on predictive-coding abnormalities in psychosis (57, 58), our model proposes that this process may increase the energetic cost and uncertainty of interoceptive inference and contribute to diminished stability of energy-demanding association networks.

A composite allostatic load index integrating cardiovascular, metabolic, endocrine, and inflammatory biomarkers has been found to be higher in first-episode psychosis and chronic schizophrenia-spectrum disorders than in healthy controls; in a meta-analysis, values were also higher in chronic samples than in first-episode samples (29, 30, 59). In some studies, a higher index was associated with greater severity of positive psychotic symptoms (29, 30) or with reduced cortical thickness (60). These findings indicate cumulative multisystem physiological burden but do not establish the direction of causality or directly measure the stress-related reallocation of energetic resources hypothesized by the present model.

Within this broader, organism-level perspective, earlier hypothesis-generating publications by Treuer and colleagues had already drawn attention to reports of hyperglycemia and insulin resistance in schizophrenia before antipsychotic treatment and proposed that metabolic, dopaminergic, gastrointestinal, immunological and infectious, and developmental mechanisms may interact in shaping vulnerability to psychosis (61, 62).

### 4.3 Gut-brain axis

In schizophrenia, the gut-brain axis can be interpreted as an immunometabolic signaling system that links peripheral state to brain function through neural, endocrine, immune, and microbial metabolite pathways. The development of the enteric nervous system (ENS) and the central nervous system (CNS) unfolds through the interaction of linked neuroectodermal and neural crest-derived developmental programs and early extrinsic vagal connections. The CNS develops from the neural tube formed during neurulation of the neural plate, whereas ENS neurons and glial cells derive predominantly from neural crest cells delaminating from the edge of the neural tube, mainly vagal and, to a lesser extent, sacral neural crest cells (63–66). Functional communication between the ENS and CNS is mediated by conserved neurotransmitter and receptor systems, autonomic/vagal reflex circuits, and endocrine, immune, and microbial/metabolite signaling mechanisms (67–69).

In mammals, microbial colonization is an evolutionarily guided process whose mechanisms can influence not only food intake, but also immunity and even the development of brain networks (70). The human gut microbiota is an important component of the gut-brain axis and can influence central nervous system function through neural (especially vagal and enteric), endocrine, immunological, and metabolic pathways.

Human studies and meta-analyses in schizophrenia indicate altered gut microbiota composition and altered inferred functional potential. However, taxon-level findings are heterogeneous, alpha diversity does not differ consistently, and lifestyle, diet, body mass index (BMI), smoking, gastrointestinal symptoms, illness stage, and antipsychotic treatment may be major confounding factors (31, 71). The issue of medication effects is especially important because antipsychotics, weight change, diet, and gastrointestinal motility can themselves modify the microbiome. Nevertheless, microbial alterations have also been reported in medication-free or first-episode drug-naive cohorts (72, 73). This picture is further informed by a two-sample, bidirectional Mendelian randomization study, which used genetic instrumental variables to suggest that a relationship compatible with causal relevance may exist between certain microbiome taxa and schizophrenia risk (74). These findings are exploratory and raise the possibility of causal relevance, but they do not identify specific microbial pathways. At present, it appears that microbiota composition and selected functional pathways may differ between a subset of patients and controls.

Among the hypothesized mechanisms, the immunological/barrier pathway is most directly compatible with immunometabolic models of schizophrenia. Altered microbiota composition may influence intestinal barrier function, translocation of bacterial components, and low-grade systemic inflammation. In schizophrenia, peripheral barrier and translocation biomarkers, as well as inflammatory cytokine patterns, provide indirect support for this route (32, 33). Inflammatory signals may influence brain function through humoral routes, effects on the blood-brain barrier endothelium, immune-cell and microglial activation, and vagal afferent sensory signaling, but the exact pathway and its specificity remain unproven.

A second important route is microbial metabolite and neurochemical signaling. Short-chain fatty acids, particularly acetate, propionate, and butyrate; metabolites of the tryptophan-kynurenine and indole pathways; bile acids; and certain neurotransmitter precursors may, in principle, influence the host organism’s immune state, gene expression, epigenetic regulation, neuroplasticity, and neurotransmission systems. In schizophrenia, metagenomic and predictive functional analyses have described alterations related to short-chain fatty acid synthesis, tryptophan metabolism, glutamatergic, GABAergic, and other neuroactive pathways (31, 72, 75).

Transplantation of microbiota from patients with schizophrenia into mice was associated with reduced hippocampal glutamate, increased glutamine and GABA, and schizophrenia-relevant behavioral abnormalities (76). These findings show that the microbiota may be capable of modulating neurochemical and behavioral phenotypes.

Overall, the gut-brain axis fits well within the immunometabolic and allostatic model of schizophrenia and is currently best understood as a modulatory system that shapes vulnerability.

### 4.4 Interoceptive timing

The gut-brain axis is a system that communicates through neural, hormonal, and immunological channels and fundamentally shapes emotional states and decision-making. The posterior-to-anterior organization of the insula may constitute a cortical interface in which viscerotopically organized interoceptive representations are integrated with salience processing, affective and motivational functions, and self-referential processing; in our model, this interface may also represent one possible neural substrate for interoceptive precision and for linking subjective timing to bodily states (77–79). Moreover, as a neuroimmune interface, the insula may link signals from the peripheral immune system with emotional and motivational processes, incentive-value processing, and visceral sensation (80).

The gastrointestinal tract (GIT) exhibits spontaneous oscillatory activity. Its electrical rhythm is generated by the pacemaker activity of interstitial cells of Cajal (ICC) located in the muscular layers of the gut, and this rhythm forms the basis of peristalsis and segmentation (81). Visceral afferent information directed toward the brain is transmitted primarily by extrinsic vagal sensory fibers. These fibers form mechano- and chemosensitive endings in the gastrointestinal mucosa and wall, and certain luminal nutrient signals may also be transmitted to vagal neurons through direct glutamatergic synaptic contacts with enteroendocrine neuropod cells (82–85).

The vagus nerve is a central component of body-brain integration (86). Visceral information mediated by vagal afferents shapes emotional appraisal, anxiety and stress responses, and modulates hippocampal memory and allostatic processes. Through responses to specific nutrients or inflammatory mediators, certain vagal neuronal subtypes, such as GLP1R+ and GPR65+ neurons, also influence motivation and reward. The vagus also plays a key role in the cholinergic anti-inflammatory reflex, which regulates immune activity and thereby shapes mood and cognitive functions over the long term, linking inflammation to emotional homeostasis (86).

In healthy participants at rest, the phase of the gastric basal rhythm (approximately 0.05 Hz), recorded with electrogastrography (EGG), was coupled to the amplitude of cortical alpha activity (10–11 Hz) measured with magnetoencephalography (MEG) (34). Using simultaneous EGG and functional magnetic resonance imaging (fMRI), Rebollo et al. (35) identified a “gastric network” characterized by phase synchronization between the gastric rhythm and regional blood-oxygen-level-dependent (BOLD) fluctuations. This network partially overlaps with conventional resting-state networks and autonomic regulatory regions and includes areas implicated in body representation and spatial processing relative to the body.

Self-experience is rooted in the inherent subjective dimension present in all experience. Its core is a pre-reflective experiential field that carries the first-personal character of experience: experiences always occur “for someone” (87). The bodily and temporal embeddedness of this self-experience is decisive. It is based on continuously updated neural maps of the internal state of the body, in which the neural representation of visceral information is central (88). The processing of external stimuli interacts with visceral sensing; the “neural subjective frame” is closely linked to homeostatic regulations involved in arousal and may connect state-consciousness with content-consciousness (88).

The neural representation of gastrointestinal functions may also be regarded as an internal metronome of interoceptive timing. If the quality of the signal or its vagal transmission is impaired by metabolic or microbial disturbance, the result may be loss of neural synchronization, timing instability, and fragmentation of interoceptive sensation.

Autonomic alterations have been documented in schizophrenia and acute psychosis, including reduced heart rate variability (HRV) and altered cardiorespiratory coupling (36, 37, 89–92). Abnormalities of gastrointestinal motility have also been reported in patients with schizophrenia (38), as well as in unaffected first-degree relatives (93). HRV provides an indirect index of cardiac vagal efferent modulation, not a direct measure of visceral vagal afferent signaling to the brain. Studies in healthy individuals have demonstrated coupling between gastric rhythms and cortical activity, including gastric phase-dependent modulation of MEG alpha amplitude and a distributed resting-state stomach–brain network (34, 35). The proposed relationship between visceral–cortical temporal coordination and self-disorder therefore represents a testable hypothesis of the present model.

### 4.5 Neotenous association networks

In the adult human brain, some regions retain juvenile developmental-biological features because neural maturation is delayed; these regions are termed “neotenous.” Evidence for prolonged synaptic development and neotenous features concerns selected cortical regions, particularly the human prefrontal cortex (40, 94, 95). Protracted neocortical myelination (39) and regional evolutionary expansion (96, 97) are distinct observations and should not be generalized to every node of the networks discussed below. Regional differences in aerobic glycolysis and cerebral energy utilization have been documented (28). Whether these metabolic characteristics confer increased vulnerability to energetic constraints in schizophrenia, particularly in late-maturing association cortical regions, is also a hypothesis-generating question.

Self-referential processing involves several interacting cortical systems rather than a single, anatomically discrete network. The default mode network (DMN), which is prominently involved in internally oriented and self-referential cognition, includes the medial prefrontal cortex, posterior cingulate cortex–precuneus, and lateral parietal regions, including the angular gyrus (98, 99). The anterior insula, in contrast, is primarily associated with the salience network, contributing to interoceptive and salience processing and to coordination between large-scale functional networks (100, 101). Self-referential functioning should therefore not be equated with a single, universally defined subdivision of the DMN.

Developmental studies have documented prolonged synaptic maturation in the human prefrontal cortex and extended trajectories of cortical myelination (39, 40, 95). These findings support the relevance of prolonged developmental plasticity in selected association cortical regions, although late maturation, evolutionary expansion, and neoteny represent distinct, potentially overlapping characteristics. Functionally, medial prefrontal and posterior cingulate–precuneus regions contribute to self-referential processing and autobiographical cognition, dorsolateral prefrontal and cingulate systems participate in cognitive control, and temporoparietal association regions contribute to social cognition and multimodal integration (99, 102–104). The possibility that the developmental characteristics of these association systems confer particular vulnerability to bioenergetic constraints in schizophrenia constitutes a hypothesis requiring direct empirical investigation.

Association cortical regions are, in fact, the neural substrates of the higher-order domains of human cognition: the networks that organize not only self-experience, but also high-level functions of predictive modeling, social understanding, and conceptual abstraction (94, 96). Among them, the medial prefrontal cortex and precuneus provide core structures for self-referential thought, introspection, and autobiographical memory (99, 105). Dorsolateral prefrontal and cingulate networks support working memory, cognitive control, and decision-making (40, 102). The temporoparietal junction, angular gyrus, and anterior insula are critical nodes of social cognition, mentalizing, language integration, and interoceptive awareness (100, 103, 104).

A substantial proportion of the structural and functional abnormalities described in schizophrenia involve frontotemporal, cingulo-insular, prefrontal, and large-scale association networks that partially overlap with late-maturing, human-specific, developmentally protracted prefrontal association systems (4, 5, 106, 107). Large-scale structural MRI meta-analysis has documented widespread cortical thinning in schizophrenia (4). In community youth with psychosis-spectrum symptoms, voxelwise MRI has identified lower regional gray-matter volumes, particularly in the bilateral medial temporal lobes (108). In typically developing children and adolescents, higher schizophrenia polygenic risk has been associated with greater cortical thickness, particularly in association areas (41). Resting-state connectivity studies and meta-analyses document dysconnectivity involving default-mode and salience-related systems, with associations between connectivity measures and cognitive performance in schizophrenia (5, 6). This interpretation is also supported by our previous subgroup-based work: after identifying neurocognitively favorable and unfavorable schizophrenia subgroups through systematic cognitive neuropsychiatric mapping (109), a subsequent voxel-based morphometry (VBM) study showed that the unfavorable (‘S’) subgroup exhibited more extensive bilateral reductions in gray-matter volume, primarily involving heteromodal and, to a lesser extent, unimodal association cortices—particularly orbitofrontal/frontopolar, cingulo-insular, subcallosal, inferior frontal, parietal opercular/supramarginal, and temporo-paralimbic regions—thereby providing subgroup-level structural evidence for the vulnerability of late-maturing associative cortical systems in schizophrenia (110).

Taken together, the findings reviewed above are compatible with the developmental vulnerability of selected integrative association networks, but do not establish a schizophrenia-specific or energetically mediated mechanism. Higher cumulative allostatic load has been associated with reduced prefrontal and parietal cortical thickness in schizophrenia (60). This association neither establishes causal directionality nor demonstrates bioenergetic mediation. Whether developmental characteristics, regional metabolic specialization, and systemic physiological burden jointly influence the stability of integrative cortical networks remains a hypothesis requiring direct investigation.

## 5 Model integration

The central hypothesis of this framework is that constraints on energetic adaptability may increase the vulnerability of temporal coordination between visceral signals and cortical activity, and disruptions of this coordination may contribute to alterations in embodied self-experience. Accordingly, a distinguishing and testable prediction of the model concerns the relationship among energetic state, directly measured visceral–cortical coupling, and independently assessed embodied self-experience.

## 6 Testable predictions

Experimentally tractable mechanistic hypotheses and their corresponding falsification criteria are summarized in **Supplementary Table S2**.

H1. Load-dependent bioenergetic vulnerability. According to the model, the mismatch between energy demand and available capacity constitutes a functionally proximal energetic constraint on adaptive brain-body regulation. At rest, the mismatch may be partially compensated for; however, in a single prespecified challenge paradigm selected on the basis of the study objective and feasibility—for example, cognitive load, psychosocial stress, sleep deprivation, inflammatory activation, or a metabolic challenge—energy-demanding networks may become more unstable. The hypothesis would be supported if metabolic indices explained variance in network and cognitive outcomes after controlling for medication exposure, BMI, smoking, sleep, and symptom severity, particularly under challenge.

H1 is intended as a modular research program. A scalable core protocol would include detailed characterization of clinical and confounding factors, fasting metabolic measurements, one prespecified challenge paradigm, and primary network and cognitive assessments. ^31^P-MRS, ^1^H-MRS, or FDG-PET could be limited to embedded subsamples tailored to the mechanistic question. Fasting metabolic measures characterize systemic metabolic status and are not substitutes for cerebral bioenergetic measurements. A null association involving these peripheral markers alone would not falsify the proposed cerebral mechanism. For example, an embedded substudy could use functional ³¹P magnetization-transfer MRS to quantify the stimulation-related change in the apparent forward creatine-kinase rate constant in an occipital region during alternating rest and sustained visual stimulation, an approach demonstrated in healthy adults at 3 T (111). This endpoint indexes activity-dependent cerebral phosphotransfer regulation rather than substrate switching or maximal energetic reserve. In studies combining H1 and H4, the relationship between energetic adaptability and challenge-related changes in directly measured visceral–cortical coupling should also be examined, using prespecified physiological measures and temporal windows.

H2. Allostatic load and interoceptive precision. The model distinguishes the energetic capacity constraint from impaired allostatic regulation. Chronically high allostatic load is expected to be associated with less accurate or more rigid interoceptive predictions, altered prediction-error weighting, and insula-salience network dysregulation. The critical claim is longitudinal: the allostatic index should predict changes in interoceptive tasks and network measures; cross-sectional co-occurrence with poorer clinical status is not sufficient.

H3. Gut-immune-metabolite subgroups. The framework does not treat the gut-brain axis as a unitary diagnostic biomarker. Rather, the microbiome, intestinal barrier, microbial metabolites, and inflammatory measures may delineate immunometabolic subgroups associated with different autonomic tone, negative or cognitive symptom profiles, and dimensions of self-experience disturbance. A stringent test requires control for diet, BMI, smoking, gastrointestinal complaints, and antipsychotic exposure, as well as the use of an independent replication sample.

H4. Interoceptive temporal coupling and self-disorder. The coupling of gastric, cardiac, and respiratory rhythms with cortical activity is proposed as a candidate correlate and potential contributor to the temporal organization of embodied self-experience. The critical prediction is not merely that EGG–EEG, ECG–EEG, or respiration–neural coupling differs in schizophrenia, but that alterations in directly measured visceral–cortical coupling are more closely associated with independently assessed disturbances of ipseity, depersonalization, bodily estrangement, and body ownership than with general psychopathological severity. These relationships should be examined after controlling for medication exposure, arousal, peripheral physiological characteristics, and other prespecified confounders. An energetic effect on embodied self-experience mediated through altered visceral–cortical coupling does not necessarily require a statistical interaction between energetic state and coupling. Joint testing with H1 would further examine whether energetic state modifies the relationship between visceral–cortical coupling and embodied self-experience. A joint test of H1 and H4 would provide a more stringent evaluation of the proposed energetic–interoceptive relationship. Four competing models should be compared in a preregistered analysis predicting independently assessed disturbances of embodied self-experience: an energetic model, an interoceptive coupling model, an additive model incorporating both domains, and an interaction model including a prespecified interaction between energetic state and directly measured visceral–cortical coupling. The incremental predictive performance of the interaction model should be evaluated against the strongest competing model in independent validation data, using a prespecified performance metric and a minimum meaningful improvement threshold. Failure to demonstrate such improvement would count against this specific moderation prediction if measurement reliability is adequate and the uncertainty interval excludes the prespecified meaningful gain. Results with insufficient measurement reliability or imprecise estimates would remain inconclusive rather than constituting falsification of the broader framework.

H5. Experimental interventions may help clarify the potential contribution of visceral–cortical temporal coordination to embodied self-experience. Transcutaneous vagus nerve stimulation (tVNS) may serve as a perturbation approach targeting putative vagal afferent pathways, whereas controlled slow breathing modifies multiple interacting cardiorespiratory, autonomic, and interoceptive processes and should therefore be investigated as a distinct, less pathway-specific intervention. Both approaches require appropriate control conditions and verification of physiological target engagement. Changes in heart rate variability alone are insufficient to establish modulation of the hypothesized central mechanism; direct measurements of visceral–cortical coupling are needed to evaluate this possibility. Acute intervention outcomes should focus on state-level embodied self-experience, assessed independently of physiological measures, and should be distinguished from more enduring basic self-disorders.

H6. Temporal ordering during intervention. One of the strongest falsification criteria for the model is temporal ordering. If energetic or interoceptive stabilization is an upstream mechanism, then physiological target engagement and normalization of network dynamics should precede sustained symptomatic or functional improvement. This would help determine whether the biomarker is a mere consequence or a mechanistic precondition of clinical change.

H7. Configurational specificity against a transdiagnostic background. The model does not assume that individual metabolic, allostatic, autonomic, or interoceptive abnormalities are specific to schizophrenia. Rather, it predicts that their combined configuration, potentially including alterations in association-network function, may characterize a subgroup within the schizophrenia spectrum. A critical test is whether these biological measures provide incremental predictive value beyond independently assessed basic self-disorder scores alone, as well as general psychopathological severity and relevant clinical confounders. This prediction should be evaluated through preregistered comparisons of models with and without biological measures, using independent validation samples and outcomes defined independently of the self-disorder assessments. Failure to demonstrate a meaningful incremental predictive gain, despite adequate measurement reliability and statistical precision, would weaken the proposed configurational specificity of the integrated model.

The seven hypotheses constitute a modular empirical testing framework rather than a universal causal or temporal sequence of illness development. Individual modules can be examined separately or in combination, allowing the proposed relationships to be evaluated against competing explanations and prespecified falsification criteria.

## 7 Alternative explanations and boundary conditions

According to the model proposed here, the converging domains delineate a vulnerability space within which schizophrenia-spectrum phenotypes may emerge under specific developmental, genetic, environmental, and treatment-related conditions. Several alternative explanations should therefore be considered. Alternative explanations, the boundary conditions of the model, and their implications for interpreting the present framework are summarized in **Supplementary Table S3**.

First, metabolic abnormalities may be consequences or amplifying factors of illness course rather than primary causes. Individuals with schizophrenia-spectrum disorders are exposed to numerous secondary metabolic risks, including antipsychotic treatment, reduced physical activity, sleep and circadian rhythm disturbances, smoking, chronic psychosocial stress, socioeconomic disadvantage, and more limited access to preventive healthcare. Network meta-analytic evidence indicates that antipsychotics differ substantially in their metabolic risk profiles (112). In addition, the elevated prevalence of metabolic syndrome is observed across several severe mental disorders, including schizophrenia-spectrum disorders, bipolar disorder, and major depression (9, 113). Lifestyle factors are also far from negligible: sedentary behavior and low physical activity are common across the full spectrum of severe mental disorders, and smoking is strongly associated with schizophrenia in international meta-analytic data (114–116). Disturbances of glucose homeostasis have been documented in first-episode, antipsychotic-naive schizophrenia and non-affective psychosis populations (12, 117), indicating that these abnormalities cannot be attributed solely to antipsychotic exposure. Possible explanations include pre-existing metabolic vulnerability, physiological and behavioral changes associated with the emerging illness, and shared antecedent factors influencing both psychosis risk and metabolic regulation. In chronic samples, metabolic abnormalities may reflect varying combinations of these factors, further influenced by medication exposure, stress-related physiological processes, lifestyle factors, and social determinants of health.

Second, microbiome findings must be interpreted cautiously because the psychiatric microbiome literature remains substantially heterogeneous. Gut microbiome composition is strongly influenced by diet, lifestyle, geography, ethnicity, medication exposure, body composition, sampling procedure, sequencing platform, and bioinformatic methodology. In psychiatric case-control studies, sample sizes are often modest, medication histories are difficult to control, and a cross-sectional design cannot determine whether microbial alterations precede illness, represent consequences of illness-associated lifestyle change, or reflect treatment-induced effects. A systematic review of major depression, bipolar disorder, and schizophrenia found no strong evidence for disorder-specific differences detectable either in alpha diversity or at the level of individual species. However, beta diversity, reflecting the patterning of entire microbial communities, appears relatively consistently different in patient groups compared with control populations without mental disorder (118). Another meta-analysis likewise identified transdiagnostic rather than disorder-specific patterns of microbiome perturbation (119). A review focused specifically on schizophrenia (120) concluded that current evidence is insufficient to determine whether the observed microbiome abnormalities are causes, consequences, correlates of external factors, or treatment-related effects. Microbiome alterations should therefore be discussed as possible modulators of immunometabolic and gut-brain signaling rather than as schizophrenia-specific biomarkers until longitudinal, medication-stratified, diet-controlled, and geographically diverse studies establish temporal precedence and diagnostic specificity.

Third, allostatic load should preferably be interpreted as a general index of cumulative multisystem stress burden. The concept of allostatic load was introduced to describe the physiological “cost” imposed by chronic adaptation to stress on neuroendocrine, immunological, cardiovascular, and metabolic systems (55, 121). This framework is highly relevant to psychosis, but by its nature it is not specific. Severe mental illnesses are chronic conditions associated with substantial functional impairment and frequently accompanied by somatic comorbidities; allostatic load has therefore been proposed as a useful framework for monitoring physiological dysregulation in these disorders (122). Elevated allostatic load may reflect chronic stress exposure, early-life adversity, inflammation, cardiometabolic burden, sleep disturbance, substance use, antipsychotic exposure, and social disadvantage, and there is still no consensus on how allostatic load should be calculated (123). In the present model, allostatic load should therefore be treated as a transdiagnostic marker of severity and chronicity that may interact with schizophrenia-spectrum vulnerability, but cannot itself be regarded as a diagnostic signature.

Fourth, the hypothesized relationship between interoception and self-disorder may also be transdiagnostic. Interoception denotes the sensing, interpretation, and integration of internal bodily signals, and altered interoceptive processing has been described in numerous mental disorders, including anxiety disorders, mood disorders, eating disorders, addictive disorders, somatic symptom disorders, and psychotic disorders (124). By contrast, the investigation of primary, basic, prereflective, or core self-experience, or ipseity disturbance, has a more specific phenomenological tradition in schizophrenia-spectrum psychopathology (1, 125). Meta-analytic evidence indicates that basic self-disorders aggregate more strongly in the schizophrenia spectrum than in other mental disorders (7). This does not mean, however, that interoceptive dysfunction itself is schizophrenia-specific, even though one study identified an association between altered interoceptive accuracy and positive psychotic-type symptomatology (126). Rather, interoceptive dysregulation may be one pathway through which bodily self-presence, agency, affective salience, and self-world boundaries become unstable. The boundary condition is therefore conceptual: interoception may contribute to self-disorder, but only a subset of interoceptive abnormalities maps onto schizophrenia-spectrum ipseity disturbance.

Fifth, the vulnerability of developmentally protracted or neotenous neural systems cannot be interpreted as a phenomenon specific to schizophrenia. Human cortical development is characterized by unusually prolonged transcriptional and synaptic maturation, especially in association and prefrontal networks (40, 127). This prolonged plasticity may create an extended vulnerability window for genetic, inflammatory, metabolic, social, and traumatic influences. At the same time, heterochrony and neoteny have also been discussed more broadly in relation to psychiatric disorders and the social brain (128), and contemporary genetic and transcriptomic findings indicate strong transdiagnostic overlap among schizophrenia, bipolar disorder, major depression, autism spectrum disorder, and other psychiatric conditions (3, 129, 130). Consequently, the model identifies neotenous or heterochrony-sensitive networks as a developmental vulnerability architecture. The diagnostic outcome probably depends on timing, cell type, affected networks, developmental stage, genetic background, sex, immunometabolic state, trauma exposure, and compensatory capacity.

## 8 Implications and future directions

Future investigations should prioritize prospectively designed multimodal studies of energetic adaptability, visceral–cortical coupling, and embodied self-experience, with appropriate consideration of medication exposure, illness stage, arousal, and peripheral physiology. Longitudinal and controlled perturbation designs may help distinguish cross-sectional associations from temporal and potentially causal relationships, while independent validation is required to establish the incremental predictive value of integrated biological measures.

Clinical implications remain provisional. Established cardiometabolic care is warranted independently of the present hypothesis, whereas interventions targeting bioenergetic adaptation, gut–brain signaling, vagal pathways, or interoceptive timing for schizophrenia-related psychopathology require controlled evaluation. Acute changes in state-level embodied self-experience should not be interpreted as evidence of improvement in more enduring basic self-disorders.

## 9 Conclusion

The present article proposes an integrative framework connecting systemic and cerebral bioenergetic alterations, predictive allostatic regulation, gut–immune–metabolite signaling, and visceral–cortical temporal coordination with developmental network characteristics and disturbances of embodied self-experience in schizophrenia-spectrum disorders. Although abnormalities within several of these domains are empirically documented, their temporal ordering and causal relationships remain unresolved. In particular, the hypothesis that limited energetic adaptability may be associated with disrupted visceral–cortical coupling and altered embodied self-experience has not yet been directly tested in schizophrenia. Neither elevated regional aerobic glycolysis nor associations between allostatic load and cortical structure establish an energetic mediating mechanism.

The distinguishing contribution of the framework is therefore a set of testable cross-domain predictions rather than an established causal explanation. Critical evaluations should combine reliable, direct measurements of visceral–cortical coupling with independently assessed self-experience, preregistered comparisons of energetic, interoceptive, additive, and integrated models, and validation in independent samples. Biological measures should also demonstrate meaningful incremental predictive value beyond basic self-disorder scores and relevant clinical covariates when configurational specificity is claimed. Adequately precise negative findings under reliable measurement conditions would weaken these specific integrative predictions, while longitudinal and perturbation-based studies are needed to clarify causal directionality. Proposed clinical applications remain hypothesis-generating pending controlled evidence of benefit.

## Data Availability

The original contributions presented in the study are included in the article/**Supplementary Material**. Further inquiries can be directed to the corresponding author.
