# Supplementary material for: The integrated bioenergetic-interoceptive model of schizophrenia-spectrum disorders
Source: Front Psychiatry. 2026 Oct 30;17:1911221. doi: 10.3389/fpsyt.2026.1911221 (PMC13760337; doi:10.3389/fpsyt.2026.1911221)
Supplement: Supplementary file 1 [file SupplementaryFile1.docx]

Supplementary Material

Supplementary Tables S1 to S3

Supplementary Table S1 presents the model architecture and evidence map; Supplementary Table S2 presents mechanistic hypotheses and falsification criteria; Supplementary Table S3 summarizes alternative explanations and boundary conditions. References S1–S76 are listed at the end of this file and are numbered independently of the main article.

# Supplementary Table S1

Architecture and evidential foundations of the integrated bioenergetic–interoceptive model of schizophrenia-spectrum disorders

## Panel A. Conceptual architecture and proposed cross-level links

| **Integration level** | **Function in the model** | **Proposed reciprocal or convergent links** | **Phenotypic relevance** | **Conceptual boundary** |
| --- | --- | --- | --- | --- |
| Energetic capacity and metabolic flexibility | Defines the relation between momentary energy demand and available capacity. Metabolic flexibility refers more narrowly to adaptive switching among substrates and energy-production pathways. | Limited capacity may increase the energetic cost of allostatic responses and reduce the precision of resource allocation. Under challenge, impaired flexibility may contribute to network instability. | Reduced cognitive efficiency, stress-related decompensation, and negative symptoms or deficit-syndrome features. | Energetic constraint is broader than metabolic flexibility and is not equivalent to metabolic syndrome. Dynamic inflexibility is a candidate mechanism, not an established uniform whole-brain phenotype. |
| Predictive allostatic regulation | Allocates metabolic, autonomic, endocrine, and immune resources in anticipation of internal and external demands. | Repeated or sustained demand with incomplete recovery may accumulate allostatic load and narrow regulatory reserve; energetic capacity and allostatic load may influence each other reciprocally. | Stress sensitivity, relapse vulnerability, affective and threat-related arousal, and salience instability. | Allostatic load is transdiagnostic. Its reciprocal energetic and interoceptive role in this framework is a model-derived, testable mechanism rather than an established linear causal pathway. |
| Gut–immune–metabolite interface | Modulates bodily input through intestinal barrier function, microbial metabolites, inflammatory tone, endocrine signaling, and vagal afferents. | Altered visceral input may modify allostatic tone and the reliability or energetic cost of brainstem-limbic-insular integration. | Biological heterogeneity, immunometabolic subgroups, and modulation of negative and cognitive symptoms, as well as anomalous experiences of the bodily self. | No unitary schizophrenia microbiome is assumed. Diet, geography, BMI, smoking, gastrointestinal function, illness stage, medication, sampling, and analysis are major sources of heterogeneity. |
| Interoceptive temporal binding | Integrates cardiac, respiratory, and gastric rhythms as candidate temporal scaffolds for embodied self-representation; the insula links visceral maps with salience, affective, motivational, and potentially immune information. | Degradation of visceral signal quality, vagal transmission, or cross-system coupling may destabilize bodily mapping, agency, and the continuity of subjective presence. | Disturbance of ipseity, depersonalization, bodily estrangement, and instability of the self-world boundary. | Autonomic-vagal dysfunction is more directly supported than gastric-brain coupling or abnormalities in insular timing in schizophrenia. The complete mechanistic chain remains hypothesis-generating. |
| Neotenous association networks | Selected late-maturing association networks retain prolonged plasticity and serve as candidate convergence sites. | Developmentally protracted systems may be especially vulnerable to interacting energetic, allostatic, inflammatory, autonomic, and interoceptive stressors. | Disturbances in self-reference, mentalizing, cognitive control, abstraction, and salience integration. | Neoteny is a transdiagnostic developmental vulnerability architecture, not a disease-specific marker. Schizophrenia-spectrum relevance is proposed to arise from the overall configuration. Late maturation, evolutionary expansion, and neoteny are distinct, potentially overlapping characteristics. High energetic demand is a model hypothesis rather than an established property of every region. |
| Clinical expression and feedback | Symptoms and functional consequences emerge from interacting vulnerabilities and feed back through behavior, sleep, treatment, lifestyle, and social context. | Positive feedback loops may increase allostatic and metabolic burden, while effective treatment may improve some endpoints and worsen others. | Chronicity, somatic comorbidity, functional decline, and long-term physical health risk. | Cross-sectional designs cannot reliably separate cause from consequence. Medication effects depend on the compound, dose, duration, and endpoint. |

Note. The model is reciprocal and nonlinear; row order does not imply a universal temporal sequence. Proposed links in Panel A are model-derived unless directly supported in Panel B. Immune-inflammatory signaling is represented within the gut–immune–metabolite interface for visual economy but is treated as a cross-level modulator.

# Supplementary Table S1 continued

## Panel B. Evidence map by model component

| **Component / evidence domain** | **Empirical basis** | **Study type, population, and treatment status** | **Diagnostic scope / directness** | **Qualitative support** | **Citations** | **Main inferential gap** |
| --- | --- | --- | --- | --- | --- | --- |
| Energetic capacity and metabolic flexibility: peripheral glucose regulation | Higher fasting glucose and insulin resistance are detectable at first episode before antipsychotic exposure. | Meta-analyses of first-episode schizophrenia and antipsychotic-naïve non-affective psychosis; participants were assessed before antipsychotic exposure. | Direct evidence from schizophrenia-spectrum samples, supplemented by broader evidence from non-affective psychosis samples. | Moderate-to-strong, convergent. | (S1, S2). | Does not establish premorbid or causal status and does not directly measure dynamic metabolic flexibility; acute illness and lifestyle remain potential confounders. |
| Energetic capacity and metabolic flexibility: cerebral glucose, lactate, and high-energy phosphate metabolism | Frontal glucose hypometabolism, elevated brain lactate, and altered creatine-kinase-mediated phosphate transfer have been reported in chronic schizophrenia and first-episode psychosis. | FDG-PET meta-analysis; in vivo ¹H-MRS, ³¹P-MRS, and ³¹P-MT-MRS; post-mortem evidence. Most samples are treated or mixed; Pettegrew et al. (S3) included 11 first-episode drug-naïve patients, whereas five of the 16 first-episode participants in Yuksel et al. (S4) were medication-free. | Direct evidence from schizophrenia samples and broader evidence from first-episode psychosis samples; limited pretreatment evidence. | Moderate support for cerebral bioenergetic abnormalities; limited evidence of independence from treatment effects. | (S3–S7). | Predominantly resting measurements; no direct test of adaptive substrate or pathway switching; regional, state, and medication effects remain. |
| Energetic capacity and metabolic flexibility: mitochondrial / OXPHOS and regional-cellular context | Mitochondrial complex-I/OXPHOS, transcriptomic, proteomic, respiratory-capacity, and ATP-related abnormalities show both regional and cellular heterogeneity, as well as cross-region convergence. | Human post-mortem multiregional, cortical-layer, and cell-type studies; iPSC-derived cells; disease-independent human brain energy maps. Post-mortem cohorts are largely chronic or have mixed treatment status; iPSC studies and healthy-brain atlas studies provide indirect evidence. | Direct evidence from schizophrenia samples, supplemented by experimental and healthy-human background evidence. | Moderate support for mitochondrial / OXPHOS dysfunction and context dependence; indirect evidence for metabolic flexibility. | (S8–S15). | Dynamic metabolic flexibility has not been measured at regional, layer, or cell-type resolution; no uniform whole-brain phenotype is established. |
| Predictive allostatic regulation: cumulative allostatic load | Composite allostatic load is elevated in first-episode and chronic psychosis or schizophrenia and has been associated with symptom severity and reduced cortical thickness. | Cross-sectional and longitudinal clinical biomarker studies and meta-analysis; early samples are not consistently drug-naïve, and chronic samples are predominantly treated or mixed. | Direct evidence from schizophrenia samples and broader evidence across psychotic disorders; the construct is transdiagnostic. | Moderate direct evidence for cumulative multisystem load. | (S16–S19). | Index construction is heterogeneous; directionality is unresolved; the index does not directly measure energetic resource reallocation. |
| Predictive allostatic regulation: predictive control and interoceptive precision | Allostasis is supported as anticipatory physiological regulation, while psychosis models implicate aberrant precision weighting and interoceptive inference. | Foundational theoretical work and experimental studies in physiology, computational neuroscience, and transdiagnostic human research; treatment status is not a defining variable. | Indirect, transdiagnostic mechanistic background. | Integrative and theory-based. | (S20–S24). | The proposed reciprocal link among energetic constraint, allostatic load, and interoceptive precision has not been directly demonstrated in schizophrenia-spectrum cohorts. |
| Gut–immune–metabolite interface: microbiome composition and functional potential | Altered microbial composition and inferred functional potential are reported, including medication-free and first-episode drug-naïve cohorts; multiomics findings implicate GABA, tryptophan, and short-chain fatty acid pathways. | Case-control 16S or metagenomic studies, systematic reviews or meta-analyses, and multiomics studies. Treated or mixed samples predominate; Zhu et al. studied medication-free patients, Ma et al. studied first-episode drug-naïve and chronic medicated groups, and Wang et al. studied first-episode drug-naïve patients. | Direct evidence from schizophrenia samples, with some evidence from broader psychosis samples. | Moderate but heterogeneous. | (S25–S30). | High sensitivity to diet, geography, BMI, smoking, gastrointestinal function, medication, sampling, and analysis; temporal precedence and a unitary disease signature are unproven. |
| Gut–immune–metabolite interface: barrier and inflammatory signaling | Altered intestinal permeability or translocation biomarkers and inflammatory profiles are consistent with a gut-immune route. | Peripheral biomarker studies in schizophrenia; samples were mostly treated or had mixed treatment status. | Direct clinical observations; indirect evidence for the proposed gut-to-brain mechanism. | Limited-to-moderate. | (S31, S32). | The relevant barrier, humoral, endothelial, microglial, or vagal route and its diagnostic specificity are not established. |
| Gut–immune–metabolite interface: microbial metabolite and neurochemical mechanisms | Predicted microbial pathways and serum metabolites involve short-chain fatty acids, tryptophan-kynurenine, GABAergic, and glutamatergic processes; patient-to-mouse microbiota transfer altered neurochemistry and behavior. | Human metagenomic or metabolomic association studies, including some drug-naïve samples, plus preclinical fecal-transfer experiments. | Direct human associative evidence plus preclinical causal support. | Moderate indirect mechanistic support. | (S25–S27, S33). | Specific human mediators, directionality, and causal relevance to symptoms or network dysfunction remain unconfirmed. |
| Interoceptive temporal binding: autonomic and vagal regulation | Reduced HF-HRV or RMSSD, baroreflex sensitivity, and cardiorespiratory coupling have been reported, including reduced parasympathetic reactivity in drug-naïve schizophrenia and first-episode drug-naïve psychosis. | HRV meta-analysis and psychophysiological case-control studies; samples included treated or mixed groups as well as direct pretreatment cohorts. | Direct evidence from schizophrenia samples, supplemented by broader evidence from first-episode psychosis samples. | Moderate, convergent. | (S34–S38). | HRV provides an indirect index of cardiac vagal efferent modulation, not a direct measure of visceral vagal afferent signaling to the brain. The findings do not directly establish gastric-brain coupling, abnormalities in insular timing, or mechanisms underlying disturbances of ipseity. |
| Interoceptive temporal binding: enteric function | Gastric dysmotility has been described in acute schizophrenia and in healthy first-degree relatives. | Small electrogastrographic or gastric-motility case-control studies; treatment status in patient samples is mixed or unclear; relatives are unmedicated. | Direct evidence from schizophrenia samples, with indirect support from familial or trait findings. | Limited but direct. | (S39, S40). | Small samples, limited replication, and no direct connection to central timing or self-disorder. |
| Interoceptive temporal binding: gastric-brain coupling and insular integration | Gastric phase modulates cortical alpha amplitude and a stomach-brain resting-state network is detectable in healthy humans; the insula integrates viscerotopic information, salience-related signals, and peripheral immune signals. | Healthy-human EGG-MEG and fMRI studies; anatomical and theoretical work; neuroimmune evidence is largely preclinical. Treatment status is not applicable to healthy cohorts. | Mechanistic background; indirect evidence for schizophrenia-spectrum disorders. | Moderate mechanistic evidence for gastric-brain coupling in healthy humans; sparse direct evidence from schizophrenia-spectrum samples; hypothesis-generating with respect to self-disorder. | (S41–S46). | No direct test in drug-naïve schizophrenia of the proposed pathway from visceral timing through insular precision to disturbance of ipseity. |
| Late-maturing association networks: developmental and regional metabolic characteristics | Selected human association cortical regions exhibit prolonged synaptic maturation and myelination. Metabolic imaging in healthy adults demonstrates elevated regional aerobic glycolysis in parts of the prefrontal and parietal cortices, without establishing increased total energy expenditure | Developmental post-mortem studies, comparative neuroanatomy, transcriptomics, and PET studies in healthy individuals. | Normative developmental and metabolic evidence; indirect relevance to schizophrenia-spectrum vulnerability | Convergent evidence for developmental and regional metabolic characteristics; indirect and hypothesis-generating evidence for energetic vulnerability in schizophrenia | (S47–S53). | Elevated regional aerobic glycolysis does not establish increased total energy demand, impaired metabolic flexibility, or greater susceptibility to energetic constraints. The proposed relationship requires direct investigation in schizophrenia-spectrum samples |
| Neotenous association networks: schizophrenia-related network abnormalities | Schizophrenia involves cortical thinning, gray-matter volume reductions, and dysconnectivity in association networks. Structural differences are also reported in community youth with psychosis-spectrum symptoms and in relation to schizophrenia polygenic risk in typically developing youth. | Large-scale structural MRI, resting-state connectivity meta-analysis, subgroup VBM, community youth imaging, and cross-sectional imaging genetics; clinical samples are mainly treated or mixed, with complementary evidence from community and typically developing cohorts. | Direct evidence from schizophrenia samples; complementary structural evidence from community youth with psychosis-spectrum symptoms and indirect developmental evidence from population imaging genetics. | Strong support for association-network abnormalities; indirect support for developmental continuity. | (S54–S58). | These cross-sectional studies do not establish developmental trajectories or bioenergetic mediation. The same cohorts rarely include metabolic, allostatic, and interoceptive measures; the proposed cross-level convergence is untested. |
| Clinical expression and feedback: cognition, self-disorder, and configurational specificity | Cognitive impairment can precede psychosis and persist, whereas basic self-disorders cluster relatively selectively within the schizophrenia spectrum and exhibit trait-like stability; interoceptive dysfunction itself is transdiagnostic. | Longitudinal studies, reviews, phenomenological and case-control studies, and meta-analyses of clinical evidence; treatment status is heterogeneous and not the principal classification variable. | Direct evidence from schizophrenia-spectrum samples for cognitive impairment and self-disorders; transdiagnostic evidence for interoceptive dysfunction. | Strong support for the phenotype; indirect evidence for the proposed integrated mechanism. | (S59–S63). | Direct links between energetic or interoceptive biomarkers and ipseity disturbance or basic self-disorders, as well as the incremental specificity of the proposed configuration, require testing. |
| Clinical expression and feedback: multisystem burden and reciprocal effects | Cardiometabolic, respiratory, and endocrine multimorbidity and reduced life expectancy are pronounced in severe mental illness; medication, sleep, lifestyle, symptoms, and social disadvantage can feed back on physiology. | Population databases, commission reports, systematic reviews, and a network meta-analysis of antipsychotics; predominantly treated or mixed clinical populations. | Direct evidence from severe mental illness and schizophrenia cohorts; not all components are disorder-specific. | Strong support for multisystem burden; limited evidence regarding the direction of feedback. | (S64–S68). | Cross-sectional and observational designs cannot separate cause, consequence, and treatment effects; repeated within-person studies are needed. |

Note to Supplementary Table A2. Qualitative support labels reflect explicit narrative judgments rather than formal GRADE ratings. Treatment status is reported using the terminology and operationalization of the original studies; antipsychotic-naïve, drug-naïve, medication-free, and minimally medicated categories are not equivalent. Classification as direct evidence refers to the sampled diagnostic population and does not by itself establish causal or temporal primacy. Abbreviations: EGG = electrogastrography; EEG = electroencephalography; FDG-PET = fluorodeoxyglucose positron-emission tomography; FEP = first-episode psychosis; HF-HRV = high-frequency heart-rate variability; iPSC = induced pluripotent stem cell; MRS = magnetic resonance spectroscopy; OXPHOS = oxidative phosphorylation; RMSSD = root mean square of successive differences. MEG = magnetoencephalography; MT-MRS = magnetization-transfer magnetic resonance spectroscopy; ATP = adenosine triphosphate; BMI = body mass index; GABA = gamma-aminobutyric acid.

# Supplementary Table S2

Experimentally tractable mechanistic hypotheses and falsification criteria derived from the proposed model

| **Hypothesis** | **Mechanistic claim** | **Proximal test / design** | **Primary outcome(s)** | **Falsification criterion** |
| --- | --- | --- | --- | --- |
| H1. Load-dependent bioenergetic vulnerability | Energetic constraint may be partly compensated at rest. Impaired metabolic flexibility is one candidate mechanism by which high-demand states reveal network vulnerability. | Modular design. Core cohort: preferably first-episode or minimally medicated participants; characterization of clinical status, medication exposure, lifestyle, and potential confounders; fasting glucose, insulin, HbA1c, and lactate; one prespecified challenge paradigm selected for the study aim; fMRI or EEG network assessment at rest and during or immediately after challenge. Nested mechanistic subsample: one advanced neuroenergetic modality (³¹P-MRS, ¹H-MRS, or FDG-PET). | Challenge-related change in DMN, SN, or FPN connectivity or modularity, and cognitive performance; modality-specific neuroenergetic measures in the nested subsample. | In an adequately powered study using the selected challenge module, metabolic measures do not predict challenge-related network instability or cognitive change after adjustment for medication exposure, BMI, smoking, sleep, and symptom severity, or the association is not stronger under or after challenge than at rest. Negative findings must be interpreted at the level of the metabolic measure used; peripheral-marker findings alone cannot falsify the cerebral bioenergetic mechanism. |
| H2. Allostatic load and interoceptive precision | Higher cumulative allostatic load prospectively predicts reduced accuracy or flexibility of interoceptive inference and altered insula-salience-network dynamics. | Six- to 12-month longitudinal cohort; repeated allostatic-load index, cortisol and inflammatory panel, interoceptive tasks with computational modeling, assessment with the EASE or another instrument for self-disorder, and insula or SN fMRI or EEG. | Change in interoceptive accuracy or precision parameters, EASE scores or other measures of self-disorder, cognition, and insula or SN dynamics. | Baseline level or change in allostatic load does not predict subsequent interoceptive or network change and does not mediate or moderate associations between energetic markers and the clinical phenotype. |
| H3. Gut–immune–metabolite subgroups | Gut–immune–metabolite profiles define reproducible immunometabolic subgroups rather than a unitary diagnostic marker. | Stool metagenomics or 16S profiling, short-chain fatty acids, tryptophan-kynurenine measures, barrier markers, CRP or IL-6, and autonomic measures; strict control for diet, BMI, smoking, gastrointestinal symptoms, and antipsychotic exposure; discovery and independent replication samples. | Replicable multimodal clusters associated with HRV or autonomic tone and with negative symptoms, cognitive impairment, or anomalous self-experience. | Clusters fail to replicate, disappear after dietary, medication, lifestyle, and technical controls, or show no association with autonomic, network, or clinical outcomes. |
| H4. Interoceptive temporal coupling and self-disorder | Coupling of gastric, cardiac, and respiratory rhythms contributes to the temporal organization of embodied self-experience. Energetic adaptability may modify the association between visceral–cortical coupling and embodied self-experience. | Simultaneous measurement using EGG, EEG, ECG, and respiratory monitoring at rest and during a body-focused task; assessment with the EASE and measures of depersonalization and body ownership; preferably with healthy and cross-diagnostic comparison groups. Preregistered comparison of energetic-only, coupling-only, additive, and interaction models, with independent validation and identical confounder adjustment. | EGG-phase-EEG-alpha-amplitude coupling, heartbeat-evoked potentials, coupling between respiration and neural activity, insula or brainstem connectivity, and their associations with self-disorder. Incremental out-of-sample predictive performance for independently assessed self-experience, relative to the best-performing simpler model. | No reproducible between-group differences in coupling and no association with self-disorder or embodied self-experience, or the associations are fully explained by general symptom severity, arousal, medication, or peripheral physiology. With adequate measurement reliability and statistical precision, the interaction model fails to achieve a prespecified minimum meaningful gain, and the uncertainty interval excludes that gain. |
| H5. Vagal modulation as a test of causal involvement | If vagal or interoceptive signal quality is causally relevant, experimentally verified short-term modulation of vagal or interoceptive signaling should alter bodily-rhythm coupling and momentary embodied self-experience. Vagal afferent and cardiorespiratory perturbations may modify visceral–cortical coupling and state-level embodied self-experience through partially distinct pathways. | Randomized, crossover, sham-controlled transcutaneous vagus nerve stimulation or coherence-based slow breathing; pre- and post-intervention EGG, EEG, ECG, and respiratory measurements; explicit verification of physiological target engagement. Separate controlled tVNS and slow-breathing protocols, with verification of physiological target engagement and simultaneous visceral and neural recordings. | HRV and vagal indices, neural coupling with gastric, cardiac, or respiratory rhythms, state-level interoceptive confidence, and measures of embodied self-experience. Directly measured visceral–cortical coupling and independently assessed momentary embodied self-experience; HRV and respiratory indices as physiological secondary outcomes. | With verified target engagement, there is no change relative to sham in coupling, interoceptive outcomes, or measures of embodied self-experience, or the observed change is unrelated to target engagement. Failure to engage the target is inconclusive rather than falsifying. With verified target engagement and adequate measurement sensitivity, neither intervention produces the prespecified meaningful changes in coupling and associated state-level embodied self-experience relative to appropriate controls. |
| H6. Temporal ordering during intervention | Within intervention studies, if energetic or interoceptive stabilization mediates improvement, physiological target engagement and network stabilization should precede sustained clinical or functional change. | Adjunctive metabolic or autonomic intervention, such as exercise, metabolic optimization, interoceptive biofeedback, or tVNS, with repeated measures at prespecified time points, for example, at weeks 0, 2, 6, and 12; time-lagged within-person models. | Temporal sequence and mediation: physiological target engagement → network stabilization → changes in cognitive performance, anomalous self-experience, or functioning. | Clinical improvement consistently precedes physiological or network change, occurs without target engagement, or time-lagged analyses show no mediation by the proposed targets. |
| H7. Configurational specificity against a transdiagnostic background | A configuration comprising energetic or allostatic features, autonomic or interoceptive features, self-disorder, and association-network features distinguishes a schizophrenia-spectrum subgroup beyond what is explained by general psychopathology. | Cross-diagnostic sample including schizophrenia-spectrum disorders, bipolar disorder, major depressive disorder, autism spectrum disorder, and trauma-related or dissociative disorders; identical protocol; preregistered multivariable or latent-profile analysis; external validation. | A validated pattern distinguishes a schizophrenia-spectrum subgroup characterized by basic self-disorders and adds predictive value beyond symptom severity, medication exposure, lifestyle variables, and diagnosis alone. Incremental out-of-sample predictive performance of biological measures beyond basic self-disorder scores and relevant clinical covariates, evaluated against independently defined schizophrenia-spectrum classification or other prespecified clinical outcomes. | The configuration provides no incremental predictive performance beyond measures of general psychopathology, medication exposure, lifestyle variables, or individual domains; the pattern does not replicate or appears identically across diagnoses. The addition of biological measures provides no meaningful improvement over a model based on self-disorder scores and relevant clinical covariates, with adequate measurement reliability and uncertainty intervals excluding the prespecified minimum meaningful predictive gain, or the observed improvement fails to replicate in independent samples. |

Note. The seven hypotheses constitute a modular empirical testing framework, not a proposed universal temporal sequence of illness development. Modules may be tested independently or combined; H6 concerns temporal ordering within intervention studies. The hypotheses, designs, outcomes, and criteria are predictions formulated by the authors on the basis of the model rather than summaries of established findings; their empirical background and evidential status are summarized in Section 4 of the main article and Supplementary Table A2. Abbreviations: BMI = body mass index; CRP = C-reactive protein; DMN = default mode network; EASE = Examination of Anomalous Self-Experience; ECG = electrocardiography; EGG = electrogastrography; EEG = electroencephalography; FDG-PET = fluorodeoxyglucose positron-emission tomography; FPN = frontoparietal network; HRV = heart-rate variability; IL-6 = interleukin 6; MRS = magnetic resonance spectroscopy; SN = salience network; tVNS = transcutaneous vagus nerve stimulation. HbA1c = glycated hemoglobin; fMRI = functional magnetic resonance imaging; MT-MRS = magnetization-transfer magnetic resonance spectroscopy.

## Clarifications for H1, H4, and H5

H1. Fasting metabolic measures characterize systemic metabolic status and are not substitutes for cerebral bioenergetic measurements. A null association involving these peripheral markers alone would not falsify the proposed cerebral mechanism. For example, an embedded substudy could use functional ³¹P magnetization-transfer MRS to quantify the stimulation-related change in the apparent forward creatine-kinase rate constant in an occipital region during alternating rest and sustained visual stimulation, an approach demonstrated in healthy adults at 3 T (S69). This endpoint indexes activity-dependent cerebral phosphotransfer regulation rather than substrate switching or maximal energetic reserve.

H4. An energetic effect on embodied self-experience mediated through altered visceral–cortical coupling does not necessarily require a statistical interaction between energetic state and coupling. Failure to demonstrate meaningful incremental prediction by the interaction model would count against this specific moderation prediction if measurement reliability is adequate and the uncertainty interval excludes the prespecified meaningful gain. Results with insufficient measurement reliability or imprecise estimates remain inconclusive rather than constituting falsification of the broader framework.

H5 clarification (Supplementary Table A3). Transcutaneous vagus nerve stimulation may target putative vagal afferent pathways, whereas controlled slow breathing modifies multiple interacting cardiorespiratory, autonomic, and interoceptive processes and is a distinct, less pathway-specific intervention. Changes in HRV alone are insufficient to establish modulation of the hypothesized central mechanism; direct measurements of visceral–cortical coupling are needed. Acute outcomes concern independently assessed state-level embodied self-experience and should be distinguished from enduring basic self-disorders.

# Supplementary Table S3

Alternative explanations, boundary conditions, and their implications for interpretation of the proposed integrative model

| **Evidence domain** | **Alternative explanation or boundary condition** | **Implication for the present model** | **Citations** |
| --- | --- | --- | --- |
| Metabolism | Effects of medication, lifestyle, illness course, and the social environment. | Metabolic abnormalities are interpreted as vulnerability or amplifying factors rather than as universally primary etiological mechanisms. Their significance is likely to depend on timing and subgroup. | (S1, S2, S64, S67, S68). |
| Microbiome | Methodological heterogeneity and sensitivity to diet, lifestyle, geography, medication, body composition, sampling, and analytical procedures. | Microbiome findings are interpreted as potential modulators and subgroup-defining features rather than as a unitary schizophrenia-specific causal or diagnostic signal. Temporal precedence requires longitudinal validation. | (S26, S29, S30, S70). |
| Allostatic load | Transdiagnostic cumulative multisystem stress burden and heterogeneity in index construction. | Allostatic load is treated as a transdiagnostic dimension of severity and chronicity that may interact with schizophrenia-spectrum vulnerability rather than as a disorder-specific diagnostic signature. | (S21, S71–S73). |
| Interoception | A transdiagnostic mechanism present across multiple forms of psychopathology. | Interoceptive dysfunction is a candidate pathway to bodily self-disorder but is not in itself specific to schizophrenia-spectrum disorders. Only a subset of interoceptive disturbances may be associated with disturbances of ipseity within the schizophrenia spectrum. | (S59, S61–S63). |
| Neoteny | Shared and transdiagnostic neurodevelopmental vulnerability. | Neoteny defines a developmental vulnerability architecture rather than a disease-specific marker. Clinical outcome is expected to depend on developmental timing, cell type, affected networks, genetic background, and interacting environmental and immunometabolic factors. | (S74–S76). |

Note. The listed citations are representative rather than exhaustive. These boundary conditions constrain the interpretation of the model and do not imply that any single alternative explanation is sufficient.

# Supplementary references

Reference labels in this list apply only to this supplementary file.

**S1.** Pillinger T, Beck K, Gobjila C, Donocik JG, Jauhar S, Howes OD. Impaired glucose homeostasis in first-episode schizophrenia: a systematic review and meta-analysis. JAMA Psychiatry (2017) 74:261–269. doi: 10.1001/jamapsychiatry.2016.3803

**S2.** Greenhalgh AM, Gonzalez-Blanco L, Garcia-Rizo C, Fernandez-Egea E, Miller BJ, Arroyo MB, et al. Meta-analysis of glucose tolerance, insulin, and insulin resistance in antipsychotic-naïve patients with nonaffective psychosis. Schizophr Res (2017) 179:57–63. doi: 10.1016/j.schres.2016.09.026

**S3.** Pettegrew JW, Keshavan MS, Panchalingam K, Strychor S, Kaplan DB, Tretta MG, et al. Alterations in brain high-energy phosphate and membrane phospholipid metabolism in first-episode, drug-naive schizophrenics: a pilot study of the dorsal prefrontal cortex by in vivo phosphorus 31 nuclear magnetic resonance spectroscopy. Arch Gen Psychiatry (1991) 48:563–568. doi: 10.1001/archpsyc.1991.01810300075011

**S4.** Yuksel C, Chen X, Chouinard VA, Nickerson LD, Gardner M, Cohen T, et al. Abnormal brain bioenergetics in first-episode psychosis. Schizophr Bull Open (2021) 2:sgaa073. doi: 10.1093/schizbullopen/sgaa073

**S5.** Du F, Cooper AJ, Thida T, Sehovic S, Lukas SE, Cohen BM, et al. In vivo evidence for cerebral bioenergetic abnormalities in schizophrenia measured using ³¹P magnetization transfer spectroscopy. JAMA Psychiatry (2014) 71:19–27. doi: 10.1001/jamapsychiatry.2013.2287

**S6.** Townsend L, Pillinger T, Selvaggi P, Veronese M, Turkheimer F, Howes O. Brain glucose metabolism in schizophrenia: a systematic review and meta-analysis of ¹⁸FDG-PET studies in schizophrenia. Psychol Med (2023) 53:4880–4897. doi: 10.1017/S003329172200174X

**S7.** Liu S, Zhang L, Fan X, Wang G, Liu Q, Yang Y, et al. Lactate levels in the brain and blood of schizophrenia patients: a systematic review and meta-analysis. Schizophr Res (2024) 264:29–38. doi: 10.1016/j.schres.2023.11.013

**S8.** Glausier JR, Enwright JF III, Lewis DA. Diagnosis- and cell type-specific mitochondrial functional pathway signatures in schizophrenia and bipolar disorder. Am J Psychiatry (2020) 177:1140–1150. doi: 10.1176/appi.ajp.2020.19111210

**S9.** Park GH, Noh H, Shao Z, Ni P, Qin Y, Liu D, et al. Activated microglia cause metabolic disruptions in developmental cortical interneurons that persist in interneurons from individuals with schizophrenia. Nat Neurosci (2020) 23:1352–1364. doi: 10.1038/s41593-020-00724-1

**S10.** Kimoto S, Hashimoto T, Berry KJ, Tsubomoto M, Yamaguchi Y, Enwright JF III, et al. Expression of actin- and oxidative phosphorylation-related transcripts across the cortical visuospatial working memory network in unaffected comparison and schizophrenia subjects. Neuropsychopharmacology (2022) 47:2061–2070. doi: 10.1038/s41386-022-01274-9

**S11.** Das SC, Hjelm BE, Rollins BL, Sequeira A, Morgan L, Omidsalar AA, et al. Mitochondria DNA copy number, mitochondria DNA total somatic deletions, Complex I activity, synapse number, and synaptic mitochondria number are altered in schizophrenia and bipolar disorder. Transl Psychiatry (2022) 12:353. doi: 10.1038/s41398-022-02127-1

**S12.** Glausier JR, Enwright JF III, Lewis DA. Cortical transcriptomic profiling reveals cellular and mitochondrial adaptive stress signatures that differ between schizophrenia and bipolar disorder. Schizophr Bull (2026) 52:sbaf147. doi: 10.1093/schbul/sbaf147

**S13.** Koopmans F, Dijkstra AA, Li WP, Klaassen RV, Gouwenberg Y, Yao S, et al. Human brain prefrontal cortex proteomics identifies compromised energy metabolism and neuronal function in schizophrenia. Nat Commun (2026) 17:2131. doi: 10.1038/s41467-026-68950-y

**S14.** Mosharov EV, Rosenberg AM, Monzel AS, Osto CA, Stiles L, Rosoklija GB, et al. A human brain map of mitochondrial respiratory capacity and diversity. Nature (2025) 641:749–758. doi: 10.1038/s41586-025-08740-6

**S15.** Pourmajidian M, Hansen JY, Shafiei G, Misic B, Dagher A. Five energy metabolism pathways show distinct regional distributions and lifespan trajectories in the human brain. PLoS Biol (2026) 24:e3003619. doi: 10.1371/journal.pbio.3003619

**S16.** Berger M, Juster RP, Westphal S, Amminger GP, Bogerts B, Schiltz K, et al. Allostatic load is associated with psychotic symptoms and decreases with antipsychotic treatment in patients with schizophrenia and first-episode psychosis. Psychoneuroendocrinology (2018) 90:35–42. doi: 10.1016/j.psyneuen.2018.02.001

**S17.** Savransky A, Chiappelli J, Fisseha F, Wisner KM, Xiaoming D, Mirmomen SM, et al. Elevated allostatic load early in the course of schizophrenia. Transl Psychiatry (2018) 8:246. doi: 10.1038/s41398-018-0299-z

**S18.** Madaria L, Aymerich C, Pedruzo B, Salazar de Pablo G, Alonso-Alconada D, Fusar-Poli P, et al. Allostatic load index across the psychosis spectrum: a systematic review and meta-analysis. Front Psychiatry (2025) 16:1590547. doi: 10.3389/fpsyt.2025.1590547

**S19.** Chiappelli J, Kochunov P, Savransky A, Fisseha F, Wisner K, Du X, et al. Allostatic load and reduced cortical thickness in schizophrenia. Psychoneuroendocrinology (2017) 77:105–111. doi: 10.1016/j.psyneuen.2016.11.021

**S20.** Sennesh E, Theriault J, Brooks D, van de Meent JW, Barrett LF, Quigley KS. Interoception as modeling, allostasis as control. Biol Psychol (2022) 167:108242. doi: 10.1016/j.biopsycho.2021.108242

**S21.** McEwen BS. Protective and damaging effects of stress mediators. N Engl J Med (1998) 338:171–179. doi: 10.1056/NEJM199801153380307

**S22.** McEwen BS, Wingfield JC. The concept of allostasis in biology and biomedicine. Horm Behav (2003) 43:2–15. doi: 10.1016/S0018-506X(02)00024-7

**S23.** Barrett LF, Quigley KS, Hamilton P. An active inference theory of allostasis and interoception in depression. Philos Trans R Soc Lond B Biol Sci (2016) 371:20160011. doi: 10.1098/rstb.2016.0011

**S24.** Sterzer P, Adams RA, Fletcher P, Frith C, Lawrie SM, Muckli L, et al. The predictive coding account of psychosis. Biol Psychiatry (2018) 84:634–643. doi: 10.1016/j.biopsych.2018.05.015

**S25.** Wang Z, Yuan X, Zhu Z, Pang L, Ding S, Li X, et al. Multiomics analyses reveal microbiome–gut–brain crosstalk centered on aberrant gamma-aminobutyric acid and tryptophan metabolism in drug-naïve patients with first-episode schizophrenia. Schizophr Bull (2024) 50:187–198. doi: 10.1093/schbul/sbad026

**S26.** Murray N, Al Khalaf S, Bastiaanssen TFS, Kaulmann D, Lonergan E, Cryan JF, et al. Compositional and functional alterations in intestinal microbiota in patients with psychosis or schizophrenia: a systematic review and meta-analysis. Schizophr Bull (2023) 49:1239–1255. doi: 10.1093/schbul/sbad049

**S27.** Zhu F, Ju Y, Wang W, Wang Q, Guo R, Ma Q, et al. Metagenome-wide association of gut microbiome features for schizophrenia. Nat Commun (2020) 11:1612. doi: 10.1038/s41467-020-15457-9

**S28.** Ma X, Asif H, Dai L, He Y, Zheng W, Wang D, et al. Alteration of the gut microbiome in first-episode drug-naive and chronic medicated schizophrenia correlate with regional brain volumes. J Psychiatr Res (2020) 123:136–144. doi: 10.1016/j.jpsychires.2020.02.005

**S29.** McGuinness AJ, Davis JA, Dawson SL, Loughman A, Collier F, O’Hely M, et al. A systematic review of gut microbiota composition in observational studies of major depressive disorder, bipolar disorder and schizophrenia. Mol Psychiatry (2022) 27:1920–1935. doi: 10.1038/s41380-022-01456-3

**S30.** Nikolova VL, Smith MRB, Hall LJ, Cleare AJ, Stone JM, Young AH. Perturbations in gut microbiota composition in psychiatric disorders: a review and meta-analysis. JAMA Psychiatry (2021) 78:1343–1354. doi: 10.1001/jamapsychiatry.2021.2573

**S31.** Gokulakrishnan K, Nikhil J, Sreeraj VS, Holla B, Thirumoorthy C, Sandhya N, et al. Altered intestinal permeability biomarkers in schizophrenia: a possible link with subclinical inflammation. Ann Neurosci (2022) 29:151–158. doi: 10.1177/09727531221108849

**S32.** Nguyen TT, Kosciolek T, Daly RE, Vázquez-Baeza Y, Swafford A, Knight R, et al. Gut microbiome in schizophrenia: altered functional pathways related to immune modulation and atherosclerotic risk. Brain Behav Immun (2021) 91:245–256. doi: 10.1016/j.bbi.2020.10.003

**S33.** Zheng P, Zeng B, Liu M, Chen J, Pan J, Han Y, et al. The gut microbiome from patients with schizophrenia modulates the glutamate-glutamine-GABA cycle and schizophrenia-relevant behaviors in mice. Sci Adv (2019) 5:eaau8317. doi: 10.1126/sciadv.aau8317

**S34.** Valkonen-Korhonen M, Tarvainen MP, Ranta-Aho P, Karjalainen PA, Partanen J, Karhu J, et al. Heart rate variability in acute psychosis. Psychophysiology (2003) 40:716–726. doi: 10.1111/1469-8986.00072

**S35.** Tai YC, Lin SH, Chen KC, Lee IH, Chen PS, Lee LT, et al. Heart rate variability with deep breathing in drug-naïve patients with schizophrenia. Appl Psychophysiol Biofeedback (2020) 45:275–282. doi: 10.1007/s10484-020-09489-6

**S36.** Clamor A, Lincoln TM, Thayer JF, Koenig J. Resting vagal activity in schizophrenia: meta-analysis of heart rate variability as a potential endophenotype. Br J Psychiatry (2016) 208:9–16. doi: 10.1192/bjp.bp.114.160762

**S37.** Peupelmann J, Boettger MK, Ruhland C, Berger S, Ramachandraiah CT, Yeragani VK, et al. Cardio-respiratory coupling indicates suppression of vagal activity in acute schizophrenia. Schizophr Res (2009) 112:153–157. doi: 10.1016/j.schres.2009.03.042

**S38.** Bär KJ, Boettger MK, Berger S, Baier V, Sauer H, Yeragani VK, et al. Decreased baroreflex sensitivity in acute schizophrenia. J Appl Physiol (2007) 102:1051–1056. doi: 10.1152/japplphysiol.00811.2006

**S39.** Peupelmann J, Quick C, Berger S, Hocke M, Tancer ME, Yeragani VK, et al. Linear and non-linear measures indicate gastric dysmotility in patients suffering from acute schizophrenia. Prog Neuropsychopharmacol Biol Psychiatry (2009) 33:1236–1240. doi: 10.1016/j.pnpbp.2009.07.007

**S40.** Berger S, Hocke M, Bär KJ. Gastric dysmotility in healthy first-degree relatives of patients with schizophrenia. Prog Neuropsychopharmacol Biol Psychiatry (2010) 34:1294–1299. doi: 10.1016/j.pnpbp.2010.07.013

**S41.** Richter CG, Babo-Rebelo M, Schwartz D, Tallon-Baudry C. Phase-amplitude coupling at the organism level: the amplitude of spontaneous alpha rhythm fluctuations varies with the phase of the infra-slow gastric basal rhythm. Neuroimage (2017) 146:951–958. doi: 10.1016/j.neuroimage.2016.08.043

**S42.** Rebollo I, Devauchelle AD, Béranger B, Tallon-Baudry C. Stomach-brain synchrony reveals a novel, delayed-connectivity resting-state network in humans. eLife (2018) 7:e33321. doi: 10.7554/eLife.33321

**S43.** Craig AD. Emotional moments across time: a possible neural basis for time perception in the anterior insula. Philos Trans R Soc Lond B Biol Sci (2009) 364:1933–1942. doi: 10.1098/rstb.2009.0008

**S44.** Kerezoudis P, Howe CL, Wu LJ, Lundstrom BN, Van Gompel JJ. Insula and the immune system: more than mere co-existence? Neurosci Bull (2022) 38:1271–1273. doi: 10.1007/s12264-022-00911-z

**S45.** Park HD, Tallon-Baudry C. The neural subjective frame: from bodily signals to perceptual consciousness. Philos Trans R Soc Lond B Biol Sci (2014) 369:20130208. doi: 10.1098/rstb.2013.0208

**S46.** Craig AD. How do you feel—now? The anterior insula and human awareness. Nat Rev Neurosci (2009) 10:59–70. doi: 10.1038/nrn2555

**S47.** Vaishnavi SN, Vlassenko AG, Rundle MM, Snyder AZ, Mintun MA, Raichle ME. Regional aerobic glycolysis in the human brain. Proc Natl Acad Sci U S A (2010) 107:17757–17762. doi: 10.1073/pnas.1010459107

**S48.** Miller DJ, Duka T, Stimpson CD, Schapiro SJ, Baze WB, McArthur MJ, et al. Prolonged myelination in human neocortical evolution. Proc Natl Acad Sci U S A (2012) 109:16480–16485. doi: 10.1073/pnas.1117943109

**S49.** Petanjek Z, Judaš M, Šimić G, Rašin MR, Uylings HBM, Rakic P, et al. Extraordinary neoteny of synaptic spines in the human prefrontal cortex. Proc Natl Acad Sci U S A (2011) 108:13281–13286. doi: 10.1073/pnas.1105108108

**S50.** Somel M, Liu X, Khaitovich P. Human brain evolution: transcripts, metabolites and their regulators. Nat Rev Neurosci (2013) 14:112–127. doi: 10.1038/nrn3372

**S51.** Hill J, Inder T, Neil J, Dierker D, Harwell J, Van Essen D. Similar patterns of cortical expansion during human development and evolution. Proc Natl Acad Sci U S A (2010) 107:13135–13140. doi: 10.1073/pnas.1001229107

**S52.** Liu X, Somel M, Tang L, Yan Z, Jiang X, Guo S, et al. Extension of cortical synaptic development distinguishes humans from chimpanzees and macaques. Genome Res (2012) 22:611–622. doi: 10.1101/gr.127324.111

**S53.** Somel M, Franz H, Yan Z, Lorenc A, Guo S, Giger T, et al. Transcriptional neoteny in the human brain. Proc Natl Acad Sci U S A (2009) 106:5743–5748. doi: 10.1073/pnas.0900544106

**S54.** van Erp TGM, Walton E, Hibar DP, Schmaal L, Jiang W, Glahn DC, et al. Cortical brain abnormalities in 4474 individuals with schizophrenia and 5098 control subjects via the Enhancing Neuro Imaging Genetics Through Meta Analysis (ENIGMA) Consortium. Biol Psychiatry (2018) 84:644–654. doi: 10.1016/j.biopsych.2018.04.023

**S55.** Dong D, Wang Y, Chang X, Luo C, Yao D. Dysfunction of large-scale brain networks in schizophrenia: a meta-analysis of resting-state functional connectivity. Schizophr Bull (2018) 44:168–181. doi: 10.1093/schbul/sbx034

**S56.** Kirschner M, Paquola C, Khundrakpam BS, Vainik U, Bhutani N, Hodzic-Santor B, et al. Schizophrenia polygenic risk during typical development reflects multiscale cortical organization. Biol Psychiatry Glob Open Sci (2023) 3:1083–1093. doi: 10.1016/j.bpsgos.2022.08.003

**S57.** Satterthwaite TD, Wolf DH, Calkins ME, Vandekar SN, Erus G, Ruparel K, et al. Structural brain abnormalities in youth with psychosis spectrum symptoms. JAMA Psychiatry (2016) 73:515–524. doi: 10.1001/jamapsychiatry.2015.3463. Erratum in: JAMA Psychiatry (2017) 74:542. doi: 10.1001/jamapsychiatry.2017.0202

**S58.** Szendi I, Szabó N, Domján N, Kincses ZT, Palkó A, Vécsei L, et al. A new division of schizophrenia revealed expanded bilateral brain structural abnormalities of the association cortices. Front Psychiatry (2017) 8:127. doi: 10.3389/fpsyt.2017.00127

**S59.** Sass LA, Parnas J. Schizophrenia, consciousness, and the self. Schizophr Bull (2003) 29:427–444. doi: 10.1093/oxfordjournals.schbul.a007017

**S60.** Sheffield JM, Barch DM. Cognition and resting-state functional connectivity in schizophrenia. Neurosci Biobehav Rev (2016) 61:108–120. doi: 10.1016/j.neubiorev.2015.12.007

**S61.** Raballo A, Poletti M, Preti A, Parnas J. The self in the spectrum: a meta-analysis of the evidence linking basic self-disorders and schizophrenia. Schizophr Bull (2021) 47:1007–1017. doi: 10.1093/schbul/sbaa201

**S62.** Khalsa SS, Adolphs R, Cameron OG, Critchley HD, Davenport PW, Feinstein JS, et al. Interoception and mental health: a roadmap. Biol Psychiatry Cogn Neurosci Neuroimaging (2018) 3:501–513. doi: 10.1016/j.bpsc.2017.12.004

**S63.** Ardizzi M, Ambrosecchia M, Buratta L, Ferri F, Peciccia M, Donnari S, et al. Interoception and positive symptoms in schizophrenia. Front Hum Neurosci (2016) 10:379. doi: 10.3389/fnhum.2016.00379

**S64.** De Hert M, Correll CU, Bobes J, Cetkovich-Bakmas M, Cohen D, Asai I, et al. Physical illness in patients with severe mental disorders. I. Prevalence, impact of medications and disparities in health care. World Psychiatry (2011) 10:52–77. doi: 10.1002/j.2051-5545.2011.tb00014.x

**S65.** Launders N, Hayes JF, Price G, Osborn DPJ. Clustering of physical health multimorbidity in people with severe mental illness: an accumulated prevalence analysis of United Kingdom primary care data. PLoS Med (2022) 19:e1003976. doi: 10.1371/journal.pmed.1003976

**S66.** Firth J, Siddiqi N, Koyanagi A, Siskind D, Rosenbaum S, Galletly C, et al. The Lancet Psychiatry Commission: a blueprint for protecting physical health in people with mental illness. Lancet Psychiatry (2019) 6:675–712. doi: 10.1016/S2215-0366(19)30132-4

**S67.** Pillinger T, McCutcheon RA, Vano L, Mizuno Y, Arumuham A, Hindley G, et al. Comparative effects of 18 antipsychotics on metabolic function in patients with schizophrenia, predictors of metabolic dysregulation, and association with psychopathology: a systematic review and network meta-analysis. Lancet Psychiatry (2020) 7:64–77. doi: 10.1016/S2215-0366(19)30416-X

**S68.** Firth J, Solmi M, Wootton RE, Vancampfort D, Schuch FB, Hoare E, et al. A meta-review of “lifestyle psychiatry”: the role of exercise, smoking, diet and sleep in the prevention and treatment of mental disorders. World Psychiatry (2020) 19:360–380. doi: 10.1002/wps.20773

**S69.** Chen C, Stephenson MC, Peters A, Morris PG, Francis ST, Gowland PA. ³¹P magnetization transfer magnetic resonance spectroscopy: Assessing the activation induced change in cerebral ATP metabolic rates at 3 T. Magn Reson Med (2018) 79:22–30. doi: 10.1002/mrm.26663

**S70.** Szeligowski T, Yun AL, Lennox BR, Burnet PWJ. The gut microbiome and schizophrenia: the current state of the field and clinical applications. Front Psychiatry (2020) 11:156. doi: 10.3389/fpsyt.2020.00156

**S71.** McEwen BS, Stellar E. Stress and the individual: mechanisms leading to disease. Arch Intern Med (1993) 153:2093–2101. doi: 10.1001/archinte.1993.00410180039004

**S72.** Bizik G, Picard M, Nijjar R, Tourjman V, McEwen BS, Lupien SJ, et al. Allostatic load as a tool for monitoring physiological dysregulations and comorbidities in patients with severe mental illnesses. Harv Rev Psychiatry (2013) 21:296–313. doi: 10.1097/HRP.0000000000000012

**S73.** Misiak B. Stress, allostatic load, and psychosis: one step forward in research but where to go next? Front Psychiatry (2020) 10:937. doi: 10.3389/fpsyt.2019.00937

**S74.** Cross-Disorder Group of the Psychiatric Genomics Consortium. Genomic relationships, novel loci, and pleiotropic mechanisms across eight psychiatric disorders. Cell (2019) 179:1469–1482.e11. doi: 10.1016/j.cell.2019.11.020

**S75.** Brüne M. Neoteny, psychiatric disorders and the social brain: hypotheses on heterochrony and the modularity of the mind. Anthropol Med (2000) 7:301–318. doi: 10.1080/713650607

**S76.** Gandal MJ, Haney JR, Parikshak NN, Leppa V, Ramaswami G, Hartl C, et al. Shared molecular neuropathology across major psychiatric disorders parallels polygenic overlap. Science (2018) 359:693–697. doi: 10.1126/science.aad6469
